# A Mendelian randomization analysis of the relationship between cardioembolic risk factors and ischemic stroke

**DOI:** 10.1038/s41598-021-93979-y

**Published:** 2021-07-16

**Authors:** Danyang Tian, Linjing Zhang, Zhenhuang Zhuang, Tao Huang, Dongsheng Fan

**Affiliations:** 1 Department of Neurology, Peking University Third Hospital, No. 49, North Garden Rd., Haidian District, Beijing, 100191 China; 2 Department of Epidemiology and Biostatistics, School of Public Health, Peking University, No. 38, Xueyuan Rd., Haidian District, Beijing, 100191 China

**Keywords:** Neurology, Risk factors

## Abstract

Observational studies have shown that several risk factors are associated with cardioembolic stroke. However, whether such associations reflect causality remains unknown. We aimed to determine whether established and provisional cardioembolic risk factors are causally associated with cardioembolic stroke. Genetic instruments for atrial fibrillation (AF), myocardial infarction (MI), electrocardiogram (ECG) indices and N-terminal pro-brain natriuretic peptide (NT-pro BNP) were obtained from large genetic consortiums. Summarized data of ischemic stroke and its subtypes were extracted from the MEGASTROKE consortium. Causal estimates were calculated by applying inverse-variance weighted analysis, weighted median analysis, simple median analysis and Mendelian randomization (MR)-Egger regression. Genetically predicted AF was significantly associated with higher odds of ischemic stroke (odds ratio (OR): 1.20, 95% confidence intervals (CI): 1.16–1.24, P = 6.53 × 10^–30^) and cardioembolic stroke (OR: 1.95, 95% CI: 1.85–2.06, P = 8.81 × 10^–125^). Suggestive associations were found between genetically determined resting heart rate and higher odds of ischemic stroke (OR: 1.01, 95% CI: 1.00–1.02, P = 0.005), large-artery atherosclerotic stroke (OR: 1.02, 95% CI: 1.00–1.04, P = 0.026) and cardioembolic stroke (OR: 1.02, 95% CI: 1.00–1.04, P = 0.028). There was no causal association of P‐wave terminal force in the precordial lead V1 (PTFVI), P-wave duration (PWD), NT-pro BNP or PR interval with ischemic stroke or any subtype.

## Introduction

Stroke leads to 10% of deaths worldwide and substantial long-term disability^1^. Cardioembolic stroke (CES) is disproportionately more disabling than nonembolic mechanisms of stroke and accounts for an increasing proportion of ischemic strokes and might multiply several-fold over the next decades in an aging society^2,3^. Furthermore, among the one-third of ischemic strokes that have no identifiable cause after standard evaluation, it is increasingly accepted that many arise from a distant embolism (cardioembolic in a large proportion) rather than in situ cerebrovascular disease. Given the heavy social and economic burden, understanding the underlying mechanisms and subsequently preventing CES are important.

Many studies have shed light on various mechanisms of suspected CES. A recent review reported that subclinical atrial fibrillation (AF), atrial cardiopathy, unrecognized myocardial infarction (MI) and patent foramen ovale are occult mechanisms inducing CES^4^. It is suspected that abnormal atrial substrate might form a nidus for thromboembolism before AF occurs^5^. Observational studies have shown associations between electrocardiographic (P-wave terminal force in lead V1 (PTFV1), P-wave duration (PWD) and maximum P-wave area^6^, biochemical (N-terminal pro-brain natriuretic peptide (NT-pro BNP))^7^, and mechanical (left atrial diameter)^8^ indicators of left atrial dysfunction and ischemic stroke risk. However, the results have not always been consistent^9^. Resting heart rate (RHR) and PR interval prolongation have been reported to be associated with AF risk, which gave us a clue to investigate whether they are associated with CES^10^. Several observational studies and meta-analyses have shown relationships between RHR and ischemic stroke, while other studies have shown inconsistent results^11^. Furthermore, the previous observational studies did not specify ischemic stroke etiologies^12^. Studies also show an association between PR interval prolongation and stroke. However, these studies contained a small number of cases. Meta-analysis of the relationship between PR interval and stroke showed negative results^13^. It is worth noting that the conclusion from observational studies may be biased due to reverse causation or confounding. Therefore, the causality behind such associations remains largely unclear.

Mendelian randomization (MR) is a technique widely used to examine the causality relationships between risk factors and various diseases in the absence of pleiotropy^14^. During conception, the genetic alleles are randomly assorted, hence, it is less likely to be affected by confounding factors comparing with observational studies. In addition, since genotype is not affected by disease, it could avoid reverse causation bias. Since the limitation of reverse causation and cofounding in observational studies, we use MR in genetic perspective, which could overcome the limitations and investigate the causal risk factors of cardioembolic stroke, and provide basis for further prevention.

## Results

**Figure 1:**
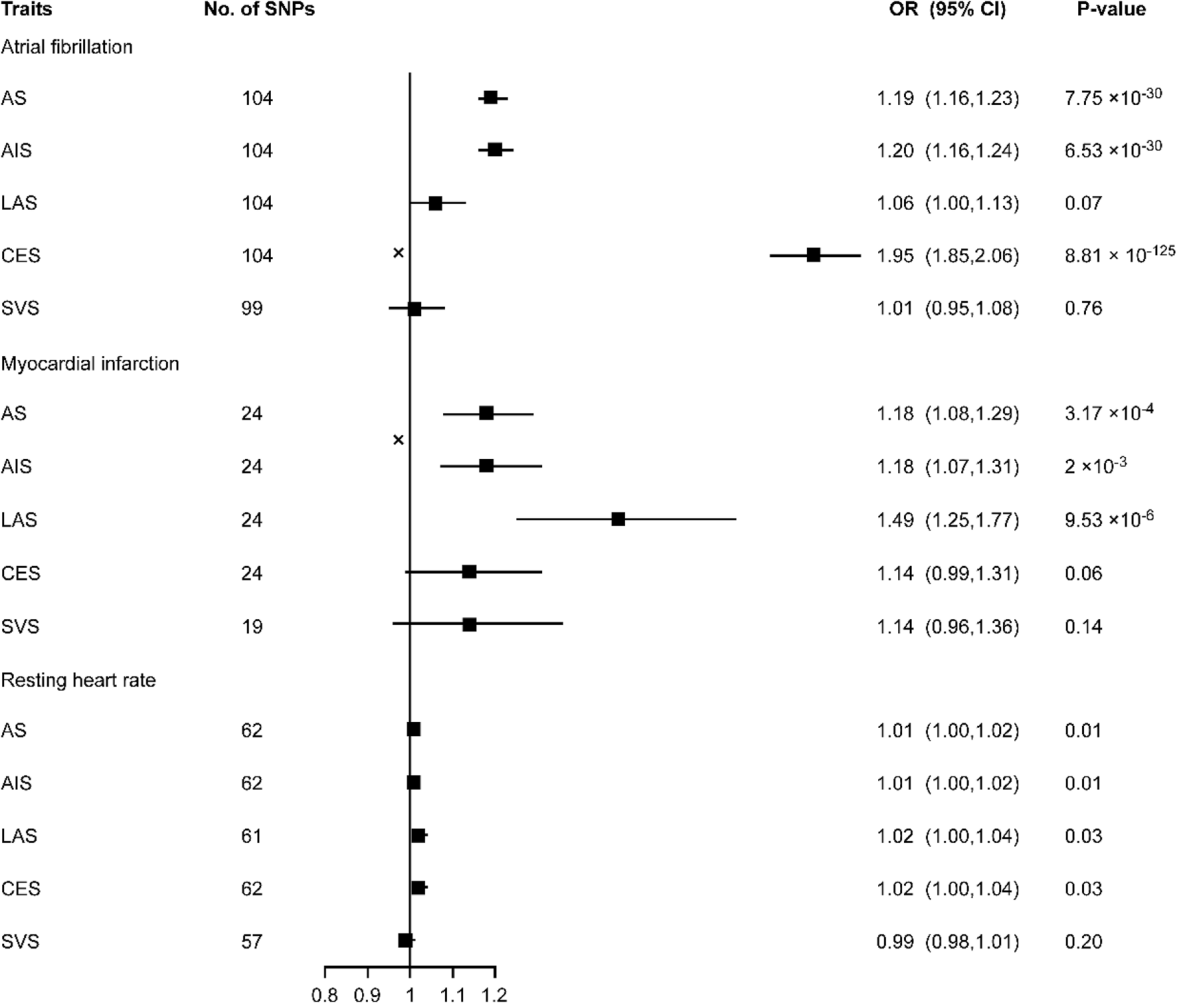
Mendelian randomization for associations between AF, MI, and RHR and stroke and its subtypes. *AF* atrial fibrillation, *MI* myocardial infarction, *RHR* resting heart rate, *SNP* single-nucleotide polymorphism, *AS* any stroke, *AIS* any ischemic stroke, *LAS* large artery atherosclerosis stroke, *CES* cardioembolic stroke, *SVS* small vessel stroke.

Genetically predicted AF was associated with significantly higher odds of any stroke (OR: 1.19, 95% CI: 1.16–1.23, P = 7.74 × 10^–30^), any ischemic stroke (OR: 1.20, 95% CI: 1.16–1.24, P = 6.53 × 10^–30^) and CES (OR: 1.95, 95% CI: 1.85–2.06, P = 8.81 × 10^–125^) but not with LAS (OR: 1.06, 95% CI: 1.00–1.13, P = 0.07) or SVS (OR: 1.01, 95% CI: 0.95–1.08, P = 0.76). (Fig. 1) The results of the leave-one-out analysis showed that no single variant had an influence on the association between AF and stroke or its subtypes. The results from the MR-Egger regression analysis, the weighted median analysis and the simple median analysis were consistent with the IVW analysis (supplement table 2). MR-Egger regression analysis provided no evidence of directional pleiotropy for the associations of AF with any stroke (intercept = 0.002, p = 0.43), any ischemic stroke (intercept = 0.002, p = 0.55) or CES (intercept = − 0.004, p = 0.39).

Genetically predicted MI was associated with significantly higher odds of any stroke (OR: 1.18, 95% CI: 1.08–1.29, P = 3.00 × 10^–4^) and LAS (OR: 1.49, 95% CI: 1.25–1.77, P = 9.53 × 10^–6^). (Fig. 1) It was suggestively associated with any ischemic stroke (OR: 1.18, 95% CI: 1.07–1.31, P = 0.002) but not with CES (OR: 1.06, 95% CI: 1.00–1.13, P = 0.06) or SVS (OR: 1.01, 95% CI: 0.95–1.08, P = 0.76). In the leave-one-out analysis, exclusion of rs1870634, rs2019090, rs2681472 rs58131196, rs1004467 or rs10176178 induced a significant result between MI and CES. The results from the weighted median analysis and the simple median analysis were consistent with the IVW analysis (supplement table 2). MR-Egger regression analysis provided no evidence of directional pleiotropy for the associations of MI with any stroke (intercept = − 0.001, p = 0.84), any ischemic stroke (intercept = − 0.005, p = 0.72) or LAS (intercept = − 0.019, p = 0.40).

A suggestively significant association was found between RHR and higher odds of any stroke (OR: 1.01, 95% CI: 1.00–1.02, P = 0.009), any ischemic stroke (OR: 1.01, 95% CI: 1.00–1.02, P = 0.005), LAS (OR: 1.02, 95% CI: 1.00–1.04, P = 0.026) and CES (OR: 1.02, 95% CI: 1.00–1.04, P = 0.028) but not with SVS (OR: 0.99, 95% CI: 0.98–1.01, P = 0.203). (Fig. 1) The results of the leave-one-out analysis showed that no single variant had an influential influence on the association between RHR and stroke or its subtypes. The results from the simple median analysis for any stroke and CES and the weighted median analysis for any stroke were consistent with the IVW analysis (supplement table 2). MR-Egger regression analysis provided no evidence of directional pleiotropy for the associations of RHR with any stroke (intercept = 6.98 × 10^–4^, p = 0.84), any ischemic stroke (intercept = 0.001, p = 0.75), LAS (intercept = − 0.004, p = 0.66) or CES (intercept = 0.004, p = 0.64).

**Figure 2:**
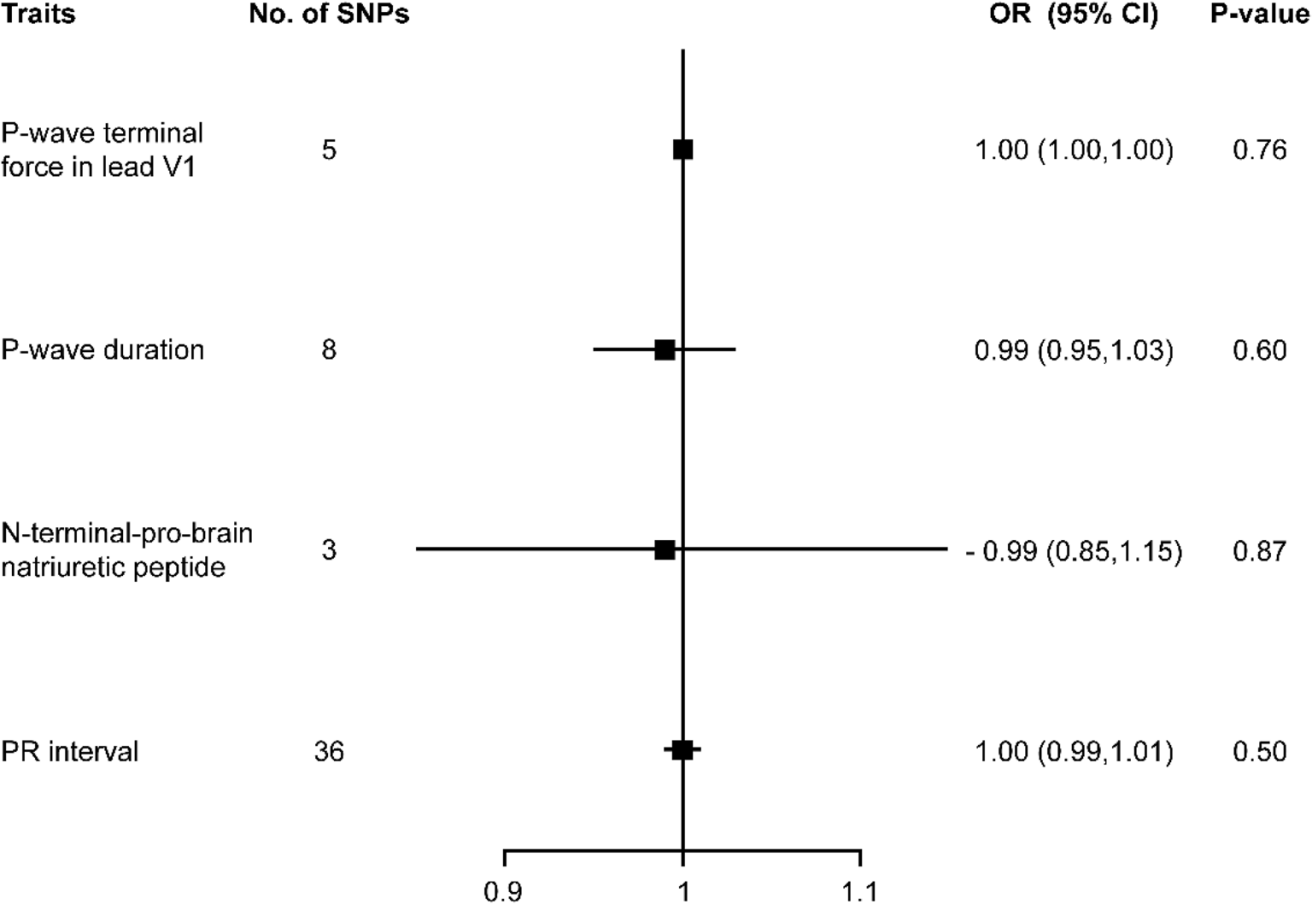
Mendelian randomization for associations between PTFVI, PWD, NT-pro BNP, and PR interval and CES. *PTFVI* P‐wave terminal force in lead V1, *PWD* P-wave duration, *CES* cardioembolic stroke.

There was no association between PTFVI, PWD, NT-pro BNP or PR interval and strokes or any subtype (Fig. 2 and supplement table 2). The results were consistent with MR-Egger regression analysis, weighted median analysis and simple median analysis, and no single genetic variant had an influential influence in the leave-one-out analyses. All MR size effect, leave one out and scatter plot results were shown in supplement figures.

## Discussion

The main findings from this study can be summarized as follows: (1) Higher odds of AF and increased RHR are causally associated with higher risk of CES; (2) We did not find evidence for a causal relationship between MI and the risk of CES unless rs1870634, rs2019090, rs2681472 rs58131196, rs1004467 or rs10176178 were excluded; (3) No evidence of causal relationship between previously observed cardioembolic risk factors such as PTFVI, PWD, NT-pro BNP or PR interval and risk of CES was found from a genetic perspective; 4. Genetically increased odds of MI and RHR are causally associated with higher risk of LAS. The present MR study explicitly examined cardioembolic-related traits while avoiding confounding and reverse causal bias.

The following implications for clinical practice are reasonable based on the main findings of this study: (1) To prevent CES and further reduce the social and economic burden, prevention of AF is an important part, which is consistent with observational studies. (2) Increasing RHR is a causal risk factor of CES, remind us to pay more attention on people with high RHR for the underlying CES burden. (3) We did not find the causal relationship between PTFVI, PWD, NT-pro BNP or PR interval and CES risk, more studies are needed, and discovering other indices for CES risk are needed.

AF is an established major risk factor for CES. This study provided causal evidence of an association between AF and CES from a genetic perspective. In addition to persistent/permanent AF, paroxysmal AF with an asymptomatic form may also cause a stroke but then revert back to sinus rhythm when the patient presents for evaluation. Detecting paroxysmal AF using prolonged heart rhythm monitoring is important to identify a stroke with cardioembolic origin^15^. Although AF is reported to be associated with atherosclerotic factors, we did not find a significant association of AF with LAS or SVS.

In observational studies, there is a high prevalence of documented stroke/transient ischemic attack (TIA) in patients with acute coronary syndromes (ACS)^16^. The risk of stroke appears to be elevated for longer than 1 month after MI^17^. Recently, it was discovered that unrecognized MIs, which make up at least one-third of all MIs, may also be a risk factor for stroke^18^. In the present MR study, we found a causal relationship between MI and ischemic stroke. However, MI was not significantly associated with CES unless rs1870634, rs2019090, rs2681472 rs58131196, rs1004467 or rs10176178 were removed. MI was identified as a cardioembolic risk factor for stroke in previous observation studies. However, evidence from this MR analysis did not support this previous conclusion. We could not exclude the possibility that the null association was due to the limited power. Another explanation is that with a larger number of variants, the potential for pleiotropy is greater, which could have diluted the association in our analysis. MI and LAS shared common atherosclerosis factors, which might indicate an association between MI and LAS. This MR study supported that MI is causally associated with the risk of LAS (P = 9.53 × 10^–6^).

The results from previous observational studies of the relationship between RHR and stroke have been inconsistent. One study has shown that when compared with patients with RHR < 65 beats/min, patients with RHR > 80 beats/min had a 38% higher risk of ischemic stroke^19^. A meta-analysis showed that the summary relative risk (RR) per 10 beats per minute increase in RHR was 1.06 (95% CI 1.02–1.10) for total stroke^20^. However, some other studies did not find an association between RHR and stroke^11^. Among previous studies, few further investigated RHR and ischemic stroke subtypes. The present MR study showed a suggested relationship between RHR and ischemic stroke, especially LAS and CES. The MR analysis could avoid confounding and reverse causal bias, which could provide evidence of causality between RHR and stroke despite the inconsistent results from observational studies. The possible mechanisms are as follows: (1) Heart rate reduction improves aortic compliance, which plays an important role in hypertension and cardiac autonomic rhythm; (2) Higher HR is related to sympathetic overactivity, which might reflect vessel stiffness, cardiac remodeling, and metabolic change and may also induce arrythmia; (3) Higher HR might induce oxidative stress, which is associated with endothelial dysfunction and promote atherosclerosis^21^.

Since cardioembolic sources are hard to detect in some cases, researchers are attempting to identify markers that suggest a cardioembolic source. The anomaly of the electrocardiographic P-wave represents abnormal atrial motion, which could induce increased atrial pressure and then electronic and mechanical remodeling^22^. The anomaly of electrocardiographic P-waves was a possible cardioembolic risk factor in previous observational studies. Previous observational studies showed inconsistent results regarding the relationship between P-wave indices (PWI) and stroke risk. A meta-analysis showed that both PTFVI and PWD were significantly associated with stroke risk. Unfortunately, the MR study did not find a relationship between PWI and stroke risk, which is consistent with some of the previous studies^23,24^. Observational studies have potential confounding factors and possible selection bias. Furthermore, we could not exclude the possibility that the present GWAS results ignored a weak association.

Previous studies have shown that patients with higher levels of NT-pro BNP have a ninefold increase in stroke risk than patients with lower NT-pro BNP levels^25^. In addition, increased NT-pro BNP levels are associated with AF risk. Even in the absence of AF, increased NT-pro BNP levels suggest atrial disorders, which contribute to stroke risk^4^. This MR study did not present a significant causal relationship between NT-pro BNP and stroke. We also could not exclude the possibility that the present GWAS results ignored a weak association. Further study is still needed.

A prolonged PR interval is thought to be associated with AF risk and pulse wave velocity, and the latter has been closely associated with the risk of LAS^26^. A prospective study showed that a PR interval > 200 ms was associated with increased stroke risk^27^. However, a meta-analysis showed that the PR interval was significantly correlated with AF and heart failure but not stroke^13^. This MR study did not find causality between the PR interval and risk of stroke.

The strengths of this study are listed below. First, the MR design technique avoids bias from reverse causation and generally reduces confounding by other modifiable environmental exposures. Second, the present study used data from large GWAS of the risk factors, which suggests strong statistical power. Third, we evaluated the causal effect of cardioembolic risk factors on ischemic stroke and its subtypes, illuminating the inconsistent results from observational studies. Fourth, we performed multiple sensitivity analyses and pleiotropy tests. These analyses showed consistent results, thus minimizing the possibility of bias in the MR analyses.

The study also has some limitations. First, the genetic variants associated with the traits explain only a small fraction of the variation in the risk factors, and we cannot exclude the possibility that the lack of significant associations of PTFV1, PWD, and NT-pro BNP with ischemic stroke subtypes was due to insufficient statistical power. Second, we did not obtain GWAS data for other cardioembolic risk factors, such as patent foramen ovale and left atrial diameter, because the data were unavailable, and further study is still needed. Third, we could not examine potential nonlinear relationships for the continuous risk factors. Finally, since the GWAS study of PR interval and NT-pro BNP were of European ethnicity, and other traits and the outcomes were transethnic, we could not avoid ethnic bias in the relationship between PR interval, NT-pro BNP and ischemic stroke.

In summary, this study provides genetic evidence that AF and RHR, but not MI may play causal roles in development of stroke, especially CES. Our findings further suggest that MI and RHR have causal relationships with LAS. PTFVI, PWD, NT-pro BNP or PR interval may not causally be associated with CES.

## Methods

### Study design

**Figure 3:**
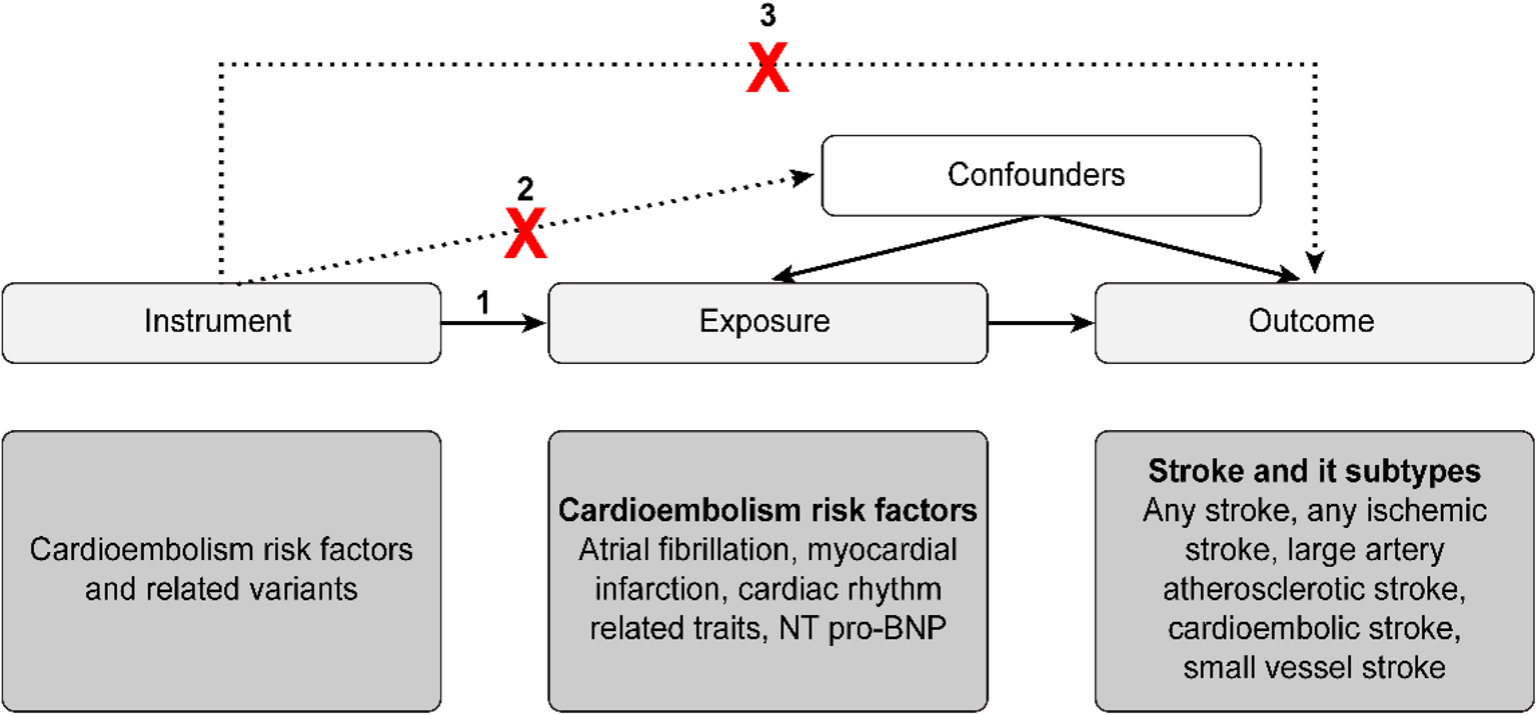
Schematic representation of Mendelian randomization analysis. Broken lines represent potential pleiotropic or direct causal effects between variables that would violate Mendelian randomization assumptions. *SNP* single-nucleotide polymorphism.

The MR approach was based on the following three assumptions (Fig. 3)^28^: first, the genetic variant selected as instrumental variable is associated with cardioembolic risk factor; second, the genetic variant is not associated with any unmeasured confounders; and third, the genetic variant is associated with ischemic stroke only through cardioembolic risk factors, not through other pathways.

### Cardioembolic risk factors

We considered cardioembolic risk factors that can be grouped under the following categories: cardiac disease related to CES, indices for left atrial abnormalities and other ECG indices that might be correlated with stroke. Within these categories, we focused on factors that were identified as having established or possible evidence for an association with ischemic stroke, especially CES.

### Data sources

Summarized data for cardioembolic risk factors (effect size estimates and their standard errors) were available from published genome-wide association studies (GWAS). We identified genetic variants with genome-wide significance (P < 5 × 10^–8^) associated with AF^29^, MI^30^, PTFV1, PWD^31^, NT-pro BNP^32^, resting heart rate (RHR)^33^ and PR interval^34^ (Table 1 and supplement Table 1).

**Table 1:** Genome-wide association studies and number of single-nucleotide polymorphisms used as instrumental variables in the Mendelian randomization analyses of cardioembolic risk factors in relationship to stroke and its subtypes.

Traits	Published GWAS				Present MR study			
	Reference	Sample size	No. of significant SNPs	r2	No. of SNPs included	Proxies used (r2 > 0.9)	Power^a^	F^a^
AF	Roselli et al., 2018^29^	65,556	104	0.15	104	0	1.00	4175
MI	Nikpay et al., 2015^30^	43,676	26	0.04	25	0	0.65	177,222
RHR	den Hoed et al., 2013^40^	134,251	68	0.02	62	0	0.06	8436
PTFV1	Christophersen et al., 2017^31^	44,456	6	0.006	5	0	0.05	2496
PWD	Christophersen et al., 2017^31^	44,456	8	0.01	8	0	0.05	4176
NT-pro BNP	Johansson et al., 2016^32^	18,624	3	0.003	3	0	0.05	1245
PR interval	van Setten et al., 2018^34^	92,340	44	0.05	36	0	0.05	21,754

*AF* atrial fibrillation, *MI *myocardial infarction, *RHR* resting heart rate, *PTFV1* P‐wave terminal force in lead V1, *PWD* P‐wave duration.

^a^Calculated for cardioembolic stroke.

For ischemic stroke and its subtypes, we collected summary statistics from the METASTROKE Collaboration^35^. GWAS from the consortiums contributed a total of 67,162 stroke cases and 454,450 controls. A total of 6688 large-artery atherosclerotic stroke (LAS)s, 9006 CESs and 11,710 small vessel strokes (SVSs) were identified using the Trial of ORG 10,172 in Acute Stroke Treatment (TOAST) classification system.

Because all analyses were based on publicly available summary statistics and not individual-level data, no patients were involved in the design of the study, and no ethical approval from an institutional review board was required.

### Genetic variants

Single-nucleotide polymorphisms (SNPs) associated with cardioembolic risk factors were selected in the combined meta-analysis of the discovery and replication samples of 6 published GWASs (Table 1). Linkage disequilibrium (defined as r^2^ < 0.01) with other genetic variants was conducted to ensure independent genetic variants. The variant with the lowest P value for association with the risk factor was selected when we encountered linkage disequilibrium. In instances where SNPs were not available in a data set because of poor imputation quality, we replaced them with proxy SNPs if available (r^2^ > 0.9).

Totally 104 SNPs were identified as associated with AF, 26 SNPs were identified as associated with MI, 68 SNPs were identified as associated with RHR, 6 SNPs were identified as associated with PTFV1, 8 SNPs were identified as associated with PWD, 3 SNPs were identified as associated with NT-pro BNP, and 44 SNPs were identified as associated with PR interval with genome wide significance (p < 5 × 10^–8^), which explained 15%, 4%, 2%, 0.6%, 1%, 0.3%, 5% variance of each exposure respectively. No linkage disequilibrium was found between SNPs. The other information of genetic variability was shown on Table 1.

### Statistical analysis

We calculated the estimates of the cardioembolic risk factors on ischemic stroke and its subtypes using the inverse-variance weighted (IVW) method, which provides a combined estimate of the causal estimate from each SNP. IVW is considered conventional MR, since it is equivalent to a two-stage least squares or allele score analysis using individual-level data^36^. Furthermore, sensitivity analyses such as the weighted median, simple median, and MR-Egger regression were conducted^37^. The results are presented as odds ratios (ORs) and 95% confidence intervals (CIs) per 10 units genetically predicted increase in each trait. The results for causal effects of diseases on cardioembolic risk factors are presented as beta ± SE per unit higher in log odds of disease. MR-Egger analysis was used to evaluate the pleiotropy effects^28^. To investigate the influence of outlying and/or pleiotropic genetic variants, we performed a leave-one-out analysis in which we omitted one genetic variant in turn^38^. The strength of the genetic instruments was tested with the F-statistic methods^39^. All SNPs for the same trait showed a strong association (F-statistic > 10).

We conducted the statistical analysis using R version 3.3.3 (R foundation). We prespecified a Bonferroni-corrected significance threshold of p = 0.00142 (where p = 0.05/35 [seven exposures and five outcomes]) to adjust for multiple testing. Associations with p values between 0.05 and 0.00142 were considered suggestive evidence of a possible association.

## Supplementary Information

Supplementary Information.
