# Supplementary material for: A Mendelian randomization analysis of the relationship between cardioembolic risk factors and ischemic stroke
Source: Sci Rep. 2021 Jul 16;11:14583. doi: 10.1038/s41598-021-93979-y (PMC8285403; doi:10.1038/s41598-021-93979-y)
Supplement: Supplementary file 1 — Supplementary Information. [file 41598_2021_93979_MOESM1_ESM.pdf]

SUPPLEMENTAL MATERIAL

Supplement table 1: Characteristics of selected SNPs.
Supplement table 2: Causal effects of stroke and its subtypes on cardioembolic risk factors.
Supplement figure1: Flowchart
Supplement figure2: MR effect size for atrial fibrillation on any ischemic stroke (AIS).
Supplement figure3: MR effect size for atrial fibrillation on cardioembolic stroke (CES).
Supplement figure4: Scatter plot for atrial fibrillation on any ischemic stroke (AIS).
Supplement figure5: Scatter plot for atrial fibrillation on cardioembolic stroke (CES).
Supplement figure6: MR effect size for myocardial infarction on any ischemic stroke (AIS).
Supplement figure7: MR effect size for myocardial infarction on large-artery atherosclerosis stroke (LAS).
Supplement figure8: Scatter plot for myocardial infarction on any ischemic stroke (QIS).
Supplement figure9: Scatter plot for myocardial infarction on large-artery atherosclerosis stroke (LAS).
Supplement figure10: leave one out analysis for myocardial infarction on cardioembolic stroke (CES).
Supplement figure11: MR effect size for resting heart rate (RHR) on any ischemic stroke (AIS).
Supplement figure12: MR effect size for resting heart rate (RHR) on large-artery atherosclerosis stroke (LAS).
Supplement figure13: MR effect size for resting heart rate (RHR) on cardioembolic stroke (CES).
Supplement figure14: Scatter plot for resting heart rate (RHR) on any ischemic stroke (AIS).
Supplement figure15: Scatter plot for resting heart rate (RHR) on large-artery atherosclerosis stroke (LAS).
Supplement figure16: Scatter plot for resting heart rate (RHR) on cardioembolic stroke (CES).

Supplement table 1: Characteristics of selected SNPs.

				risk factor			AS			AIS			LAS			CES			SVS		
	SNP	Chr	Nearest gene	EA	β	SE	β	SE	P	β	SE	P	β	SE	P	β	SE	P	β	SE	P
Atrial Fibrillation	rs10165883	2	SNRNP27	C	0.068	0.007	-0.012	0.008	0.134	-0.010	0.009	0.235	-0.037	0.020	0.069	-0.048	0.017	0.005	-0.020	0.017	0.253
Atrial Fibrillation	rs10213171	4	ARHGAP10	G	0.104	0.014	-0.012	0.015	0.428	-0.010	0.015	0.510	-0.025	0.040	0.525	-0.059	0.034	0.085	0.011	0.031	0.717
Atrial Fibrillation	rs1044258	10	C10orf76	T	0.049	0.007	-0.010	0.009	0.241	-0.011	0.009	0.222	-0.017	0.022	0.433	-0.036	0.018	0.051	-0.034	0.019	0.064
Atrial Fibrillation	rs10520260	4	HAND2	A	0.058	0.007	0.020	0.010	0.032	0.022	0.010	0.021	0.019	0.023	0.422	0.046	0.019	0.018	0.011	0.019	0.581
Atrial Fibrillation	rs10753933	1	PPFIA4	T	0.077	0.007	0.030	0.008	0.000	0.027	0.009	0.002	0.019	0.021	0.375	0.071	0.018	0.000	0.023	0.018	0.196
Atrial Fibrillation	rs10760361	9	PSMB7	G	0.039	0.007	-0.019	0.008	0.022	-0.020	0.009	0.020	-0.042	0.022	0.051	-0.055	0.018	0.002	-0.019	0.018	0.300
Atrial Fibrillation	rs10842383	12	LINC00477,BCAT1	C	0.104	0.011	-0.001	0.011	0.952	0.001	0.012	0.910	0.072	0.029	0.011	-0.040	0.024	0.099	-0.012	0.024	0.619
Atrial Fibrillation	rs10873299	14	LRRC 74, IRF2BPL	A	0.049	0.010	0.010	0.008	0.219	0.011	0.009	0.217	0.001	0.022	0.949	0.013	0.018	0.475	0.021	0.018	0.238
Atrial Fibrillation	rs11001667	10	C10orf11	G	0.058	0.007	-0.007	0.010	0.469	-0.004	0.010	0.724	-0.015	0.025	0.533	-0.058	0.022	0.008	0.005	0.020	0.803
Atrial Fibrillation	rs11180703	12	KRR1,PHLDA1	G	0.049	0.007	-0.007	0.008	0.391	-0.011	0.009	0.224	-0.011	0.021	0.604	-0.013	0.018	0.479	-0.013	0.018	0.482
Atrial Fibrillation	rs11264280	1	KCNN3,PMVK	T	0.131	0.007	0.004	0.009	0.661	-0.002	0.010	0.862	-0.080	0.025	0.001	0.048	0.020	0.014	0.000	0.021	0.990
Atrial Fibrillation	rs113819537	12	SSPN	C	0.049	0.010	-0.003	0.009	0.715	-0.004	0.009	0.677	0.035	0.023	0.130	0.004	0.020	0.849	0.004	0.019	0.853
Atrial Fibrillation	rs11598047	10	NEURL	G	0.157	0.011	-0.023	0.011	0.031	-0.022	0.012	0.060	-0.017	0.028	0.553	-0.109	0.023	0.000	0.040	0.025	0.101
Atrial Fibrillation	rs11768850	7	SUN1	T	0.039	0.005	0.028	0.008	0.001	0.029	0.009	0.001	0.040	0.020	0.048	0.032	0.018	0.064	0.037	0.017	0.027
Atrial Fibrillation	rs11773845	7	CAV1	A	0.113	0.007	0.015	0.008	0.057	0.014	0.008	0.108	0.001	0.020	0.967	0.084	0.017	0.000	0.009	0.017	0.582
Atrial Fibrillation	rs11773884	7	CDK6	A	0.049	0.010	0.035	0.009	0.000	0.034	0.009	0.000	0.003	0.023	0.903	0.036	0.019	0.058	0.035	0.019	0.063
Atrial Fibrillation	rs117984853	6	UST	T	0.113	0.014	0.020	0.017	0.238	0.038	0.018	0.039	0.076	0.047	0.104	0.065	0.036	0.072			
Atrial Fibrillation	rs12044963	1	KCND3	T	0.077	0.012	-0.004	0.012	0.749	0.000	0.012	0.972	0.002	0.028	0.934	0.063	0.027	0.018	-0.030	0.022	0.173
Atrial Fibrillation	rs12208899	6	LINC00326,EYA4	A	0.049	0.010	0.001	0.011	0.964	-0.001	0.011	0.899	0.030	0.026	0.246	0.056	0.022	0.011	-0.024	0.022	0.264
Atrial Fibrillation	rs12298484	12	DNAH10	C	0.049	0.007	-0.005	0.009	0.547	-0.009	0.009	0.309	-0.035	0.022	0.116	-0.022	0.018	0.224	-0.030	0.018	0.110
Atrial Fibrillation	rs12591736	15	TLE3,UACA	G	0.058	0.010	-0.009	0.011	0.435	-0.009	0.012	0.437	-0.029	0.027	0.289	-0.006	0.025	0.807	0.060	0.021	0.004
Atrial Fibrillation	rs12809354	12	PKP2	C	0.077	0.012	-0.012	0.011	0.304	-0.012	0.012	0.296	-0.004	0.030	0.883	-0.047	0.025	0.057	0.026	0.025	0.306
Atrial Fibrillation	rs12810346	12	TBX5-AS1, TBX3	T	0.068	0.010	-0.005	0.014	0.737	-0.004	0.015	0.805	0.045	0.037	0.230	0.015	0.029	0.600	0.024	0.034	0.480

Atrial Fibrillation	rs12908004	15	LINC00927,ARNT2	G	0.077	0.009	-0.030	0.011	0.007	-0.031	0.012	0.010	-0.016	0.030	0.590	-0.081	0.024	0.001	-0.066	0.026	0.011
Atrial Fibrillation	rs12908437	15	IGF1R	T	0.049	0.007	0.006	0.008	0.430	0.005	0.009	0.555	0.008	0.020	0.708	0.038	0.017	0.028	-0.014	0.017	0.405
Atrial Fibrillation	rs12992412	2	MBD5	T	0.039	0.007	-0.007	0.008	0.362	-0.005	0.009	0.587	-0.011	0.020	0.601	-0.013	0.018	0.456	-0.025	0.017	0.134
Atrial Fibrillation	rs1307274	6	C6orf1,NUDT3	T	0.077	0.014	0.005	0.016	0.750	0.011	0.016	0.481	0.001	0.040	0.991	0.035	0.036	0.331	-0.057	0.031	0.070
Atrial Fibrillation	rs13191450	6	GJA1,HSF2	A	0.068	0.007	0.025	0.008	0.004	0.027	0.009	0.003	-0.006	0.022	0.798	0.067	0.018	0.000	0.007	0.018	0.695
Atrial Fibrillation	rs146518726	1	C1orf185	A	0.166	0.026	-0.012	0.029	0.681	-0.008	0.031	0.800	0.043	0.087	0.618	0.146	0.067	0.030			
Atrial Fibrillation	rs17079881	6	SLC35F1	G	0.086	0.009	-0.005	0.012	0.652	-0.002	0.013	0.852	-0.034	0.031	0.278	-0.031	0.025	0.229	0.004	0.028	0.897
Atrial Fibrillation	rs174048	5	ARHGAP26,NR3C1	C	0.068	0.010	0.013	0.012	0.259	0.022	0.013	0.077	0.013	0.031	0.687	-0.015	0.026	0.562	0.044	0.027	0.098
Atrial Fibrillation	rs17490701	3	PHLDB2	G	0.068	0.012	-0.004	0.012	0.741	-0.007	0.013	0.584	0.030	0.032	0.345	-0.022	0.027	0.401	0.033	0.029	0.262
Atrial Fibrillation	rs1822273	11	NAV2	G	0.068	0.010	-0.029	0.009	0.001	-0.030	0.009	0.002	-0.022	0.023	0.336	-0.047	0.020	0.021	0.001	0.018	0.947
Atrial Fibrillation	rs210632	1	UBE4B	A	0.049	0.010	0.009	0.009	0.306	0.006	0.010	0.578	0.016	0.023	0.478	0.023	0.020	0.267	-0.008	0.019	0.661
Atrial Fibrillation	rs2129977	4	PITX2,C4orf32	A	0.399	0.009	0.073	0.009	0.000	0.077	0.010	0.000	-0.003	0.023	0.892	0.277	0.020	0.000	0.013	0.019	0.488
Atrial Fibrillation	rs2145274	20	CASC20,BMP2	A	0.104	0.014	0.010	0.015	0.511	0.011	0.016	0.508	0.009	0.042	0.823	0.009	0.033	0.797	-0.025	0.037	0.504
Atrial Fibrillation	rs2145587	14	AKAP6	A	0.077	0.009	0.006	0.009	0.475	0.008	0.009	0.415	0.042	0.023	0.066	0.033	0.020	0.099	0.002	0.019	0.922
Atrial Fibrillation	rs2286466	16	RPS2	G	0.068	0.010	-0.004	0.011	0.701	-0.003	0.011	0.759	0.020	0.027	0.463	-0.053	0.023	0.023	-0.002	0.022	0.939
Atrial Fibrillation	rs2306272	3	LRIG1	C	0.049	0.007	0.004	0.009	0.666	0.004	0.009	0.643	0.066	0.023	0.003	-0.040	0.020	0.044	0.009	0.018	0.609
Atrial Fibrillation	rs2359171	16	ZFHX3	A	0.191	0.008	0.045	0.010	0.000	0.045	0.010	0.000	0.051	0.025	0.038	0.176	0.021	0.000	0.029	0.020	0.137
Atrial Fibrillation	rs2540949	2	CEP68	A	0.077	0.007	0.022	0.008	0.006	0.025	0.009	0.004	0.014	0.020	0.499	0.032	0.017	0.065	0.027	0.017	0.115
Atrial Fibrillation	rs2738413	14	SYNE2	A	0.077	0.007	0.008	0.008	0.340	0.009	0.009	0.322	0.022	0.021	0.284	0.061	0.017	0.000	-0.022	0.017	0.192
Atrial Fibrillation	rs2834618	21	LOC100506385	T	0.113	0.011	0.011	0.013	0.420	0.015	0.014	0.292	-0.037	0.034	0.279	0.104	0.030	0.000	-0.014	0.030	0.642
Atrial Fibrillation	rs28631169	14	MYH7	T	0.068	0.010	-0.014	0.011	0.198	-0.007	0.012	0.529	-0.072	0.030	0.017	0.031	0.023	0.176	-0.052	0.027	0.055
Atrial Fibrillation	rs295114	2	SPATS2L	C	0.068	0.010	-0.012	0.008	0.137	-0.015	0.009	0.088	-0.006	0.021	0.767	-0.064	0.018	0.000	0.021	0.017	0.219
Atrial Fibrillation	rs3176326	6	CDKN1A	G	0.058	0.010	-0.048	0.010	0.000	-0.051	0.011	0.000	-0.025	0.027	0.347	-0.096	0.023	0.000	-0.016	0.023	0.472
Atrial Fibrillation	rs34750263	5	WNT8A,NME5	T	0.086	0.007	0.005	0.009	0.527	0.005	0.009	0.601	-0.003	0.022	0.875	0.040	0.019	0.030	-0.006	0.018	0.727
Atrial Fibrillation	rs34969716	6	KDM1B	A	0.086	0.009	0.021	0.010	0.032	0.025	0.010	0.017	-0.015	0.025	0.563	0.035	0.021	0.097	0.035	0.021	0.093
Atrial Fibrillation	rs35006907	8	MTSS1, LINC0096	A	0.049	0.007	0.007	0.009	0.436	0.009	0.009	0.305	-0.001	0.022	0.962	0.011	0.019	0.566	-0.006	0.018	0.739

Atrial Fibrillation	rs35349325	12	BEST3	T	0.049	0.007	0.003	0.008	0.765	0.008	0.009	0.395	0.019	0.021	0.384	0.043	0.018	0.014	0.009	0.018	0.630
Atrial Fibrillation	rs35504893	2	TTN	T	0.086	0.007	0.020	0.009	0.035	0.020	0.010	0.039	0.019	0.023	0.422	0.036	0.021	0.081	0.008	0.019	0.672
Atrial Fibrillation	rs3822259	4	WDR1	T	0.049	0.007	0.022	0.008	0.010	0.022	0.009	0.012	0.025	0.021	0.234	0.015	0.018	0.431	0.038	0.017	0.029
Atrial Fibrillation	rs3960788	4	SLC9B1	C	0.049	0.007	-0.006	0.008	0.496	-0.007	0.009	0.386	0.001	0.020	0.953	-0.038	0.017	0.028	0.026	0.017	0.117
Atrial Fibrillation	rs4385527	9	C9orf3	A	0.095	0.007	0.012	0.008	0.142	0.013	0.009	0.145	0.013	0.021	0.527	0.003	0.018	0.869	0.024	0.017	0.158
Atrial Fibrillation	rs4484922	1	CASQ2	G	0.068	0.007	-0.010	0.009	0.268	-0.008	0.009	0.398	-0.028	0.023	0.222	0.020	0.019	0.295	-0.039	0.020	0.052
Atrial Fibrillation	rs4743034	9	ZNF462	A	0.049	0.010	0.001	0.009	0.949	0.004	0.010	0.702	0.011	0.026	0.671	-0.024	0.020	0.229	0.016	0.023	0.496
Atrial Fibrillation	rs4855075	3	GNB4	T	0.058	0.010	-0.021	0.010	0.033	-0.022	0.011	0.039	0.074	0.025	0.003	-0.092	0.023	0.000	-0.009	0.020	0.649
Atrial Fibrillation	rs4951261	1	NUCKS1	C	0.049	0.007	0.000	0.011	0.980	0.002	0.012	0.874	0.075	0.029	0.009	-0.044	0.026	0.099	-0.007	0.025	0.763
Atrial Fibrillation	rs4977397	9	SLC24A2,MLLT3	A	0.039	0.007	0.041	0.010	0.000	0.048	0.011	0.000	0.070	0.025	0.005	0.107	0.022	0.000	-0.004	0.020	0.851
Atrial Fibrillation	rs55734480	7	DGKB	A	0.049	0.010	0.007	0.010	0.480	0.002	0.010	0.863	0.022	0.026	0.403	0.060	0.021	0.003	-0.030	0.023	0.188
Atrial Fibrillation	rs55754224	4	CAMK2D	T	0.049	0.010	0.067	0.014	0.000	0.070	0.015	0.000	0.078	0.038	0.039	0.067	0.030	0.025			
Atrial Fibrillation	rs55985730	7	OPN1SW	G	0.095	0.019	-0.001	0.012	0.911	-0.012	0.012	0.312	0.013	0.031	0.665	-0.032	0.026	0.211	-0.017	0.025	0.500
Atrial Fibrillation	rs56181519	2	WIPF1 ,CHRNA1	C	0.077	0.009	-0.008	0.027	0.771	0.003	0.028	0.931	-0.037	0.073	0.614	0.063	0.056	0.261			
Atrial Fibrillation	rs60212594	10	SYNPO2L	G	0.113	0.011	-0.021	0.008	0.008	-0.025	0.009	0.004	0.016	0.021	0.438	-0.087	0.018	0.000	-0.008	0.017	0.647
Atrial Fibrillation	rs608930	1	GORAB,PRRX1	G	0.095	0.007	-0.017	0.008	0.028	-0.019	0.008	0.021	-0.004	0.020	0.826	-0.067	0.017	0.000	0.014	0.017	0.385
Atrial Fibrillation	rs62011291	15	USP3	G	0.049	0.007	0.026	0.011	0.024	0.035	0.012	0.003	0.027	0.029	0.350	0.009	0.023	0.688	0.044	0.028	0.107
Atrial Fibrillation	rs62483627	7	COG5	A	0.049	0.010	-0.040	0.018	0.021	-0.034	0.019	0.073	-0.012	0.049	0.804	-0.017	0.039	0.652	-0.087	0.043	0.045
Atrial Fibrillation	rs62521286	8	FBX032	G	0.122	0.014	0.012	0.008	0.128	0.017	0.009	0.057	0.003	0.021	0.872	-0.003	0.017	0.849	0.029	0.017	0.092
Atrial Fibrillation	rs6462078	7	CREB5	A	0.058	0.010	0.005	0.009	0.571	0.008	0.010	0.420	-0.020	0.024	0.394	0.043	0.022	0.044	-0.009	0.019	0.650
Atrial Fibrillation	rs6546620	2	KIF3C	C	0.068	0.010	0.002	0.008	0.779	0.006	0.009	0.490	0.010	0.022	0.645	-0.004	0.018	0.839	0.038	0.019	0.043
Atrial Fibrillation	rs6742276	2	XP01	A	0.049	0.007	0.007	0.008	0.405	0.010	0.009	0.271	0.016	0.021	0.435	-0.006	0.018	0.731	0.033	0.018	0.062
Atrial Fibrillation	rs6790396	3	SCN10A	G	0.068	0.007	-0.002	0.009	0.796	0.003	0.009	0.722	-0.006	0.022	0.767	-0.040	0.018	0.028	0.051	0.018	0.004
Atrial Fibrillation	rs6810325	3	CAND2	C	0.077	0.007	-0.004	0.010	0.738	-0.004	0.011	0.732	-0.026	0.027	0.350	-0.024	0.024	0.314	0.008	0.022	0.719
Atrial Fibrillation	rs6882776	5	NKX2-5	G	0.058	0.007	-0.015	0.007	0.040	-0.015	0.008	0.067	-0.004	0.020	0.849	-0.029	0.017	0.083	-0.023	0.016	0.171
Atrial Fibrillation	rs6907805	6	CGA,ZNF292	G	0.039	0.007	0.015	0.008	0.074	0.012	0.009	0.159	0.012	0.021	0.577	0.029	0.018	0.111	0.016	0.018	0.378

Atrial Fibrillation	rs6993266	8	PTK2	A	0.049	0.007	-0.007	0.008	0.413	-0.002	0.009	0.846	0.019	0.021	0.368	0.015	0.018	0.404	-0.016	0.017	0.356
Atrial Fibrillation	rs716845	5	KCNN2	A	0.058	0.010	-0.019	0.011	0.084	-0.029	0.012	0.014				-0.075	0.024	0.002	0.021	0.026	0.415
Atrial Fibrillation	rs7219869	17	KCNJ2,CASC17	G	0.049	0.007	-0.022	0.008	0.009	-0.018	0.009	0.044	-0.033	0.021	0.115	-0.030	0.020	0.125	-0.035	0.017	0.039
Atrial Fibrillation	rs7269123	20	C20orf166	C	0.049	0.007	0.002	0.010	0.864	0.002	0.010	0.838	0.007	0.023	0.759	-0.026	0.020	0.205	-0.004	0.018	0.847
Atrial Fibrillation	rs72700114	1	METTL11B,LINC01142	C	0.199	0.015	0.054	0.016	0.001	0.057	0.018	0.001	0.058	0.045	0.198	0.165	0.035	0.000	0.022	0.040	0.581
Atrial Fibrillation	rs72811294	17	MYOCD	G	0.068	0.010	0.001	0.013	0.928	-0.002	0.013	0.877	0.002	0.033	0.953	-0.050	0.029	0.078	-0.005	0.028	0.845
Atrial Fibrillation	rs72926475	2	REEP1,KDM3A	G	0.068	0.012	-0.022	0.013	0.092	-0.022	0.014	0.109	-0.004	0.035	0.907	-0.044	0.028	0.113	0.025	0.031	0.431
Atrial Fibrillation	rs73032363	3	THRB	A	0.039	0.007	0.010	0.009	0.278	0.003	0.009	0.716	0.022	0.022	0.314	0.005	0.019	0.777	0.006	0.018	0.746
Atrial Fibrillation	rs73241997	14	SNX6, CFL2	T	0.068	0.012	0.006	0.010	0.547	0.006	0.011	0.609	0.009	0.027	0.751	0.040	0.023	0.082	0.006	0.022	0.777
Atrial Fibrillation	rs73366713	6	ATXN1	G	0.104	0.011	-0.014	0.013	0.276	-0.019	0.014	0.178	-0.029	0.035	0.419	-0.037	0.028	0.185	0.039	0.031	0.215
Atrial Fibrillation	rs74022964	15	HCN4,REC114	T	0.104	0.009	0.011	0.011	0.334	0.020	0.013	0.122	-0.048	0.030	0.114	0.062	0.024	0.011	-0.023	0.027	0.398
Atrial Fibrillation	rs7460121	8	MIR30B	A	0.068	0.012	0.016	0.015	0.286	0.009	0.016	0.594	-0.027	0.039	0.486	0.052	0.032	0.103			
Atrial Fibrillation	rs74910854	7	GTF2I	G	0.095	0.014	-0.016	0.024	0.489	-0.015	0.024	0.552	-0.096	0.058	0.098	0.017	0.047	0.721	-0.007	0.051	0.886
Atrial Fibrillation	rs7508	8	ASAH1	A	0.068	0.007	0.016	0.009	0.067	0.013	0.009	0.166	0.012	0.022	0.577	0.046	0.020	0.021	-0.017	0.018	0.337
Atrial Fibrillation	rs76097649	11	KCNJ5	A	0.122	0.016	0.015	0.017	0.390	0.029	0.017	0.095	0.022	0.046	0.633	0.098	0.036	0.007	-0.019	0.039	0.618
Atrial Fibrillation	rs7632427	3	EPHA3	T	0.039	0.007	0.002	0.008	0.794	0.005	0.009	0.554	-0.019	0.022	0.378	0.041	0.018	0.024	-0.019	0.019	0.326
Atrial Fibrillation	rs76774446	17	GOSR2	A	0.068	0.012	0.053	0.013	0.000	0.064	0.014	0.000	0.025	0.036	0.492	0.122	0.028	0.000	0.044	0.034	0.188
Atrial Fibrillation	rs7789146	7	KCNH2	G	0.058	0.010	-0.017	0.010	0.096	-0.018	0.011	0.092	0.017	0.025	0.503	-0.032	0.022	0.146	0.002	0.020	0.903
Atrial Fibrillation	rs7846485	8	XP07	C	0.086	0.012	-0.005	0.012	0.689	-0.004	0.012	0.771	-0.029	0.030	0.336	-0.054	0.026	0.034	-0.012	0.024	0.622
Atrial Fibrillation	rs79187193	1	GJA5	G	0.113	0.018	-0.029	0.020	0.153	-0.031	0.022	0.150	0.003	0.054	0.953	-0.092	0.044	0.034			
Atrial Fibrillation	rs7919685	10	REEP3	G	0.058	0.007	0.007	0.008	0.379	0.004	0.009	0.645	0.002	0.020	0.906	-0.017	0.017	0.326	0.044	0.017	0.009
Atrial Fibrillation	rs7978685	12	NACA	T	0.058	0.007	0.005	0.009	0.612	0.008	0.010	0.413	-0.019	0.023	0.408	0.009	0.020	0.638	0.004	0.019	0.853
Atrial Fibrillation	rs8073937	17	POLR2A, TNFSF12	G	0.049	0.007	-0.018	0.008	0.033	-0.020	0.009	0.024	-0.015	0.021	0.463	-0.056	0.018	0.002	-0.024	0.017	0.156
Atrial Fibrillation	rs880315	1	CASZ1	C	0.039	0.007	-0.053	0.008	0.000	-0.052	0.009	0.000	-0.018	0.022	0.418	-0.044	0.018	0.016	-0.085	0.018	0.000
Atrial Fibrillation	rs883079	12	TBX5	T	0.122	0.007	0.023	0.009	0.007	0.030	0.009	0.001	0.011	0.022	0.598	0.083	0.019	0.000	0.016	0.018	0.367
Atrial Fibrillation	rs949078	11	sorli,mirioohG	C	0.049	0.007	-0.025	0.009	0.005	-0.023	0.010	0.018	-0.024	0.023	0.301	-0.024	0.020	0.234	-0.010	0.019	0.608

Atrial Fibrillation	rs9580438	13	LINC00540,BASP1P1	C	0.058	0.007	-0.008	0.009	0.345	-0.007	0.009	0.480	-0.002	0.023	0.936	-0.050	0.019	0.008	-0.007	0.020	0.718
Atrial Fibrillation	rs9872035	3	PAK2	C	0.039	0.007	-0.007	0.008	0.430	-0.008	0.009	0.377	0.004	0.022	0.857	-0.049	0.018	0.006	0.019	0.018	0.293
Atrial Fibrillation	rs9953366	18	SMAD7	C	0.049	0.007	-0.029	0.009	0.001	-0.030	0.009	0.001	-0.016	0.022	0.468	-0.028	0.019	0.140	-0.018	0.018	0.324
Myocardial Infarction	rs1004467	10	CYP17A1-CNNM2-NT5C2	A	0.077	0.015	-0.003	0.012	0.801	-0.006	0.012	0.616	0.058	0.029	0.045	-0.040	0.026	0.127	-0.035	0.022	0.117
Myocardial Infarction	rs10176176	2	VAMP5-VAMP8-GGCX	T	0.068	0.010	0.010	0.008	0.218	0.012	0.008	0.157	-0.010	0.020	0.631	0.018	0.018	0.315	0.012	0.017	0.486
Myocardial Infarction	rs10455872	6	LPA	G	0.285	0.033	-0.032	0.020	0.098	-0.039	0.021	0.065	-0.238	0.053	0.000	-0.042	0.043	0.333			
Myocardial Infarction	rs11556924	7	ZC3HC1	C	0.068	0.013	-0.009	0.010	0.383	-0.006	0.011	0.575	-0.040	0.028	0.150	0.015	0.021	0.499	0.006	0.024	0.799
Myocardial Infarction	rs1332329	6	KCNK5	C	0.077	0.010	-0.021	0.008	0.013	-0.025	0.009	0.006	-0.070	0.022	0.001	-0.017	0.018	0.350	-0.021	0.018	0.233
Myocardial Infarction	rs1544935	2	AK097927	T	0.077	0.013	0.023	0.010	0.018	0.019	0.011	0.070	0.018	0.026	0.493	0.010	0.022	0.630	-0.024	0.021	0.258
Myocardial Infarction	rs180803	22	POM121L9P *	G	0.191	0.036	-0.063	0.024	0.009	-0.070	0.024	0.004	-0.093	0.053	0.078	-0.067	0.058	0.246	-0.107	0.038	0.005
Myocardial Infarction	rs1870634	10	CXCL12	G	0.068	0.010	-0.001	0.009	0.948	0.005	0.009	0.611	-0.007	0.023	0.771	0.041	0.019	0.028	0.007	0.018	0.708
Myocardial Infarction	rs2019090	11	PDGFD	A	0.068	0.010	-0.012	0.009	0.153	-0.013	0.009	0.158	0.012	0.022	0.594	-0.030	0.019	0.111			
Myocardial Infarction	rs2315065	6	PLG	A	0.262	0.030	0.034	0.019	0.068	0.052	0.020	0.010	0.230	0.051	0.000	0.038	0.042	0.361			
Myocardial Infarction	rs2505083	10	KIAA1462	C	0.058	0.010	-0.024	0.009	0.007	-0.027	0.009	0.005	-0.057	0.023	0.012	-0.034	0.018	0.060	-0.017	0.020	0.394
Myocardial Infarction	rs2681472	12	ATP2B1	G	0.077	0.013	0.035	0.010	0.001	0.038	0.011	0.000	0.046	0.025	0.062	0.031	0.022	0.169	0.009	0.020	0.655
Myocardial Infarction	rs28451064	21	KCNE2 (gene desert)	A	0.122	0.020	-0.026	0.014	0.067	-0.030	0.015	0.048	-0.117	0.040	0.003	0.035	0.030	0.242			
Myocardial Infarction	rs2891168	9	9p21	G	0.191	0.010	-0.043	0.008	0.000	-0.043	0.008	0.000	-0.073	0.020	0.000	-0.009	0.019	0.654	-0.033	0.017	0.052
Myocardial Infarction	rs35700460	1	MIA3	G	0.086	0.013	-0.012	0.009	0.191	-0.013	0.010	0.192	-0.033	0.023	0.158	-0.022	0.021	0.298	-0.018	0.018	0.325
Myocardial Infarction	rs532436	9	ABO	A	0.113	0.013	0.007	0.009	0.467	0.006	0.010	0.568	-0.063	0.024	0.007	0.033	0.020	0.105	0.002	0.020	0.933
Myocardial Infarction	rs55791371	19	LDLR	A	0.104	0.020	-0.027	0.009	0.004	-0.024	0.010	0.014	-0.012	0.024	0.632	-0.003	0.020	0.881	-0.107	0.021	0.000
Myocardial Infarction	rs55940034	13	COL4A1/A2	G	0.068	0.013	-0.060	0.020	0.003	-0.070	0.022	0.001	0.007	0.057	0.905	-0.119	0.043	0.006			
Myocardial Infarction	rs56131196	19	APOC1	A	0.086	0.015	-0.011	0.010	0.256	-0.008	0.010	0.468	0.022	0.026	0.393	-0.061	0.021	0.004	-0.023	0.024	0.336
Myocardial Infarction	rs653178	12	SH2B3	C	0.077	0.010	0.007	0.010	0.526	0.008	0.011	0.452	0.023	0.027	0.378	-0.024	0.023	0.285	0.006	0.022	0.795
Myocardial Infarction	rs7165042	15	ADAMTS7	C	0.058	0.013	0.016	0.009	0.092	0.014	0.010	0.165	0.032	0.024	0.180	0.050	0.020	0.013	0.006	0.021	0.766
Myocardial Infarction	rs72689147	4	GUCY1A3	G	0.077	0.013	-0.029	0.010	0.004	-0.030	0.011	0.005	0.013	0.025	0.615	-0.006	0.022	0.801	-0.022	0.021	0.290
Myocardial Infarction	rs7528419	1	SORT1	A	0.104	0.013	0.026	0.010	0.008	0.023	0.011	0.033	0.078	0.026	0.003	0.011	0.022	0.614	0.024	0.023	0.304

Myocardial Infarction	rs9349379	6	PHACTR1	G	0.131	0.010	0.005	0.009	0.595	0.011	0.009	0.232	0.015	0.022	0.509	-0.033	0.019	0.080	0.029	0.019	0.125
Myocardial Infarction	rs9970807	1	PPAP2B	C	0.113	0.020	-0.023	0.015	0.118	-0.019	0.016	0.217	-0.028	0.040	0.484	0.024	0.032	0.456	0.002	0.035	0.952
PR interval	rs11067773	12	MED13L	C	-1.300	0.230	-0.021	0.014	0.135	-0.029	0.015	0.050	-0.057	0.035	0.102	-0.057	0.031	0.062	-0.038	0.029	0.183
PR interval	rs11264339	1	KRTCAP2	T	-0.700	0.110	-0.005	0.009	0.568	-0.006	0.009	0.514	-0.007	0.021	0.745	-0.035	0.018	0.045	0.012	0.018	0.515
PR interval	rs11465506	14	IL25/MYH6	A	-6.400	1.040	-0.015	0.045	0.739				0.094	0.129	0.468	-0.059	0.105	0.571			
PR interval	rs11708996	3	SCN5A	C	3.100	0.180	-0.004	0.013	0.737	0.002	0.013	0.893	-0.007	0.033	0.840	0.037	0.027	0.172	0.021	0.029	0.467
PR interval	rs11763856	7	TBX20/HERPUD2	T	3.100	0.490	0.036	0.032	0.266	0.049	0.034	0.155	-0.108	0.093	0.241	0.106	0.071	0.135			
PR interval	rs12127701	1	MYBPHL	G	1.700	0.280	-0.013	0.017	0.454	-0.002	0.018	0.914	-0.054	0.050	0.277	0.011	0.039	0.788			
PR interval	rs12257568	10	SH3PXD2A/OBFC1	T	1.000	0.120	0.029	0.008	0.000	0.026	0.009	0.002	0.041	0.021	0.056	0.029	0.018	0.108	0.062	0.017	0.000
PR interval	rs12359272	10	ALDH18A1/SORBS1	A	1.000	0.130	0.016	0.009	0.074	0.016	0.010	0.108	0.014	0.024	0.556	-0.007	0.018	0.708	0.039	0.021	0.066
PR interval	rs12678719	8	ZFPM2	G	0.800	0.130	0.018	0.009	0.045	0.018	0.009	0.054	0.011	0.022	0.617	0.033	0.019	0.085	-0.010	0.018	0.577
PR interval	rs13018106	2	FIGN	C	-0.800	0.120	0.006	0.008	0.433	0.002	0.008	0.822	0.014	0.020	0.499	-0.019	0.017	0.265	-0.002	0.017	0.931
PR interval	rs13087058	3	PDZRN3	C	-1.000	0.120	0.001	0.009	0.920	-0.003	0.010	0.733	-0.017	0.024	0.487	-0.011	0.019	0.553	0.015	0.021	0.483
PR interval	rs1372797	11	NAV2	T	-1.100	0.180	0.023	0.011	0.031	0.024	0.011	0.031	0.024	0.026	0.366	0.001	0.025	0.971	0.027	0.021	0.187
PR interval	rs16858828	3	PHLDB2	C	0.900	0.150	0.001	0.011	0.956	0.005	0.012	0.686	-0.006	0.031	0.851	-0.012	0.024	0.629	-0.014	0.028	0.620
PR interval	rs17287293	12	C12orf67/SOX5	G	-2.200	0.160	0.000	0.012	0.981	-0.002	0.013	0.865	-0.070	0.030	0.018	0.053	0.027	0.047	0.015	0.025	0.545
PR interval	rs17446418	4	CAMK2D	G	0.800	0.130	-0.010	0.010	0.326	-0.006	0.011	0.598	-0.014	0.026	0.598	-0.068	0.021	0.001	0.016	0.023	0.494
PR interval	rs17767398	14	SNORD56B SIPA1L1	G	1.000	0.130	-0.005	0.010	0.580	-0.001	0.010	0.929	0.002	0.026	0.949	-0.011	0.021	0.585	-0.045	0.022	0.037
PR interval	rs1896312	12	TBX3	C	1.600	0.130	0.012	0.009	0.164	0.013	0.009	0.159	-0.003	0.022	0.908	0.024	0.020	0.220	0.022	0.018	0.222
PR interval	rs1984481	17	MYOCD	C	-0.800	0.120	-0.005	0.008	0.520	-0.008	0.009	0.350	0.007	0.021	0.735	-0.012	0.018	0.496	-0.010	0.017	0.563
PR interval	rs2129561	7	MKLN1	A	-1.000	0.120	-0.022	0.008	0.007	-0.023	0.009	0.011	-0.030	0.021	0.154	-0.016	0.018	0.377	-0.017	0.018	0.329
PR interval	rs255292	5	BNIP1/NKX2-5	C	-1.100	0.120	0.011	0.008	0.179	0.013	0.009	0.144	-0.043	0.021	0.045	0.055	0.018	0.002	0.005	0.018	0.793
PR interval	rs2585897	13	XPO4	A	1.200	0.150	-0.008	0.010	0.452	-0.004	0.011	0.685	0.024	0.026	0.353	-0.028	0.023	0.228	0.012	0.021	0.575
PR interval	rs2732860	2	TMEM182	G	-0.900	0.110	0.001	0.008	0.885	0.005	0.008	0.557	0.012	0.021	0.579	0.007	0.017	0.675	-0.003	0.017	0.840
PR interval	rs343849	4	ARHGAP24	A	-2.100	0.130	0.012	0.009	0.205	0.018	0.010	0.061	0.029	0.023	0.204	0.036	0.021	0.091	0.032	0.020	0.109
PR interval	rs3733409	4	FAT1	T	0.900	0.170	0.000	0.011	0.999	-0.001	0.011	0.958	-0.029	0.027	0.271	-0.032	0.024	0.191	0.000	0.021	0.993

PR interval	rs3807989	7	CAV1/CAV2	A	2.000	0.120	-0.019	0.008	0.020	-0.017	0.009	0.047	-0.005	0.020	0.803	-0.091	0.017	0.000	-0.005	0.017	0.789
PR interval	rs3856447	2	ID2	A	1.200	0.110	0.011	0.008	0.198	0.009	0.009	0.313	0.022	0.021	0.301	-0.011	0.017	0.516	-0.003	0.017	0.860
PR interval	rs397637	1	OBSCN	T	0.800	0.120	-0.002	0.009	0.858	-0.006	0.009	0.485	-0.011	0.022	0.610	0.000	0.021	0.998	-0.029	0.018	0.115
PR interval	rs4430933	2	MEIS1	A	1.300	0.110	-0.008	0.012	0.481	-0.011	0.013	0.389	-0.054	0.031	0.081	-0.016	0.024	0.512			
PR interval	rs4648819	1	SKI	G	-1.700	0.280	0.009	0.009	0.354	0.009	0.010	0.345	0.026	0.023	0.270	0.050	0.021	0.014	-0.013	0.019	0.500
PR interval	rs4901308	14	FERMT2	T	-0.800	0.150	0.008	0.008	0.348	-0.001	0.009	0.929	0.009	0.020	0.666	0.005	0.017	0.782	0.002	0.017	0.916
PR interval	rs6441111	3	CCNL1	C	0.800	0.130	0.005	0.010	0.592	-0.001	0.011	0.936	-0.002	0.027	0.947	0.013	0.021	0.550	-0.049	0.024	0.041
PR interval	rs6489953	12	TBX5	C	1.200	0.150	-0.002	0.010	0.818	-0.006	0.011	0.611	0.035	0.028	0.207	-0.019	0.021	0.369			
PR interval	rs652673	11	WNT11	C	-0.800	0.150	-0.063	0.009	0.000	-0.077	0.010	0.000	-0.094	0.026	0.000	-0.058	0.020	0.003	-0.104	0.023	0.000
PR interval	rs6599250	3	SCN10A	T	3.800	0.110	0.012	0.008	0.153	0.013	0.009	0.127	0.025	0.021	0.216	0.054	0.018	0.002	0.004	0.017	0.821
PR interval	rs718426	13	EFHA1	G	-1.200	0.110	0.010	0.008	0.245	0.008	0.009	0.397	0.031	0.021	0.144	0.009	0.018	0.613	-0.029	0.018	0.102
PR interval	rs7538988	1	EPS15	C	-2.100	0.370	0.012	0.028	0.663	0.009	0.030	0.772	-0.064	0.086	0.453	-0.208	0.063	0.001			
PR interval	rs7638853	3	SENP2	A	-0.700	0.120	0.013	0.008	0.130	0.016	0.009	0.071	-0.016	0.021	0.447	-0.018	0.019	0.334	0.028	0.018	0.118
PR interval	rs7729395	5	PAM	T	2.400	0.370	0.028	0.023	0.224	0.018	0.025	0.467	0.040	0.069	0.563	0.094	0.053	0.073			
PR interval	rs881301	8	FGFR1	C	0.800	0.120	0.001	0.008	0.942	0.001	0.009	0.954	0.021	0.021	0.308	0.007	0.017	0.702	0.013	0.017	0.440
PR interval	rs900669	3	FRMD4B	A	0.800	0.130	0.009	0.009	0.317	0.010	0.009	0.279	-0.003	0.023	0.910	-0.002	0.020	0.918	0.014	0.019	0.458
PR interval	rs904974	15	TLE3	T	1.100	0.190	-0.017	0.011	0.130	-0.015	0.012	0.216	-0.009	0.029	0.764	-0.015	0.025	0.537	-0.038	0.024	0.125
PR interval	rs922984	2	TTN	T	1.500	0.230	-0.013	0.012	0.291	-0.013	0.013	0.296	0.021	0.030	0.484	0.003	0.028	0.921	-0.007	0.023	0.780
PR interval	rs9590974	13	LRCH1	C	1.100	0.120	0.028	0.008	0.001	0.029	0.009	0.001	0.049	0.021	0.020	0.013	0.018	0.474	-0.014	0.017	0.418
PR interval	rs9826413	3	EOMES	T	2.000	0.360	0.001	0.018	0.951	0.001	0.019	0.953	0.037	0.048	0.451	-0.009	0.041	0.824	0.029	0.038	0.449
P-wave duration	rs11894252	2	EPAS1	T	0.520	0.090	-0.010	0.008	0.239	-0.005	0.009	0.590	-0.018	0.022	0.410	0.028	0.017	0.095	-0.018	0.019	0.348
P-wave duration	rs1467026	3	CAND2	G	0.510	0.090	0.001	0.008	0.940	-0.006	0.009	0.512	-0.003	0.021	0.905	0.029	0.018	0.110	-0.028	0.017	0.106
P-wave duration	rs3801995	7	CAV1/CAV2	T	0.600	0.090	-0.017	0.009	0.051	-0.014	0.009	0.130	0.006	0.023	0.775	-0.085	0.020	0.000	-0.005	0.019	0.804
P-wave duration	rs41312411	3	SCN5A	G	1.900	0.140	-0.004	0.012	0.759	-0.012	0.013	0.359	-0.004	0.032	0.910	-0.040	0.026	0.127	-0.011	0.028	0.700
P-wave duration	rs4276421	5	HCN1	C	0.580	0.080	0.018	0.008	0.029	0.016	0.009	0.075	0.067	0.021	0.002	0.071	0.018	0.000	-0.037	0.018	0.039
P-wave duration	rs452036	14	MYH6	A	0.640	0.090	-0.008	0.008	0.363	-0.011	0.009	0.225	-0.013	0.021	0.527	-0.017	0.018	0.334	-0.026	0.017	0.127

P-wave duration	rs562408	1	SSBP3	G	0.520	0.090	-0.013	0.009	0.133	-0.008	0.009	0.370	-0.012	0.022	0.595	-0.028	0.018	0.131	-0.014	0.018	0.459
P-wave duration	rs7312625	12	TBX5	G	0.800	0.090	0.022	0.009	0.011	0.030	0.009	0.001	0.011	0.022	0.632	0.081	0.020	0.000	0.029	0.019	0.129
P-wave terminal force	rs11242779	6	C6orf195	T	72.000	12.000	-0.011	0.008	0.172	-0.010	0.009	0.247	-0.035	0.021	0.105	0.002	0.018	0.934	0.017	0.018	0.347
P-wave terminal force	rs2115630	15	ALPK3/NMB	T	85.000	14.000	0.000	0.008	0.990	-0.002	0.008	0.793	0.008	0.021	0.717	-0.015	0.017	0.399	-0.016	0.018	0.382
P-wave terminal force	rs4435363	19	PPP5D1	A	96.000	16.000	-0.008	0.010	0.456	-0.005	0.011	0.638	-0.031	0.027	0.246	0.010	0.022	0.655	-0.026	0.024	0.285
P-wave terminal force	rs445754	14	MYH6	T	136.000	14.000	-0.002	0.008	0.818	-0.003	0.009	0.749	-0.001	0.021	0.977	-0.038	0.019	0.043	-0.015	0.017	0.384
P-wave terminal force	rs4839185	1	KCND3	C	117.000	13.000	0.009	0.012	0.437	0.016	0.012	0.201	-0.016	0.030	0.600	0.023	0.026	0.373	0.014	0.025	0.581
Resting Heart Rate	rs1050288	12	KLHL42n	T	0.213	0.036	0.023	0.009	0.009	0.026	0.009	0.005	0.017	0.022	0.445	0.029	0.019	0.140	0.020	0.018	0.271
Resting Heart Rate	rs10739663	9	MAPKAP1ne	G	0.266	0.033	-0.002	0.008	0.789	0.001	0.008	0.894	-0.024	0.020	0.227	-0.005	0.017	0.760	0.005	0.017	0.778
Resting Heart Rate	rs10841486	12	PDE3And	C	0.238	0.040	0.019	0.011	0.074	0.016	0.012	0.156	0.023	0.029	0.430	0.005	0.023	0.843	0.022	0.026	0.402
Resting Heart Rate	rs10880689	12	ALG10Bn	A	0.208	0.034	0.022	0.009	0.017	0.028	0.010	0.006	0.015	0.025	0.548	0.048	0.020	0.016	0.006	0.022	0.774
Resting Heart Rate	rs11081761	18	GAREMn	A	0.267	0.047	0.017	0.010	0.093	0.014	0.011	0.182	0.008	0.026	0.755	0.040	0.024	0.091	0.009	0.020	0.650
Resting Heart Rate	rs11083258	18	CDH2nd	C	0.276	0.045	-0.010	0.012	0.402	-0.002	0.013	0.894	-0.060	0.031	0.055	-0.019	0.025	0.463	0.024	0.029	0.413
Resting Heart Rate	rs11563648	7	ZNF800n	C	0.231	0.037	0.000	0.010	0.970	-0.003	0.010	0.782	-0.003	0.025	0.890	-0.030	0.020	0.137	-0.010	0.022	0.667
Resting Heart Rate	rs11920570	3	CCDC58n	G	0.268	0.037	-0.012	0.009	0.180	-0.023	0.010	0.017	-0.030	0.023	0.185	-0.052	0.020	0.009	-0.028	0.019	0.145
Resting Heart Rate	rs12501032	4	PPARGC1An	C	0.288	0.036	0.002	0.010	0.843	0.006	0.011	0.582	-0.014	0.026	0.609	-0.013	0.021	0.545	0.013	0.024	0.581
Resting Heart Rate	rs12576326	11	TP53I11n	A	0.253	0.036	0.007	0.009	0.453	0.009	0.010	0.336	-0.004	0.023	0.870	0.011	0.020	0.587	0.000	0.019	0.993
Resting Heart Rate	rs12579753	12	PPFIA2ne	T	0.246	0.039	0.002	0.010	0.866	-0.002	0.011	0.873	0.008	0.027	0.757	-0.010	0.022	0.645	0.003	0.023	0.901
Resting Heart Rate	rs12713404	2	BCL11An	T	0.199	0.035	-0.003	0.008	0.711	0.000	0.009	0.993	0.006	0.021	0.782	-0.008	0.018	0.669	-0.005	0.017	0.753
Resting Heart Rate	rs12721051	19	APOEn; APOC1n; PVRL2d	G	0.287	0.044	-0.003	0.012	0.812	0.008	0.013	0.524	-0.032	0.032	0.314	0.027	0.026	0.303	0.002	0.026	0.942
Resting Heart Rate	rs12889267	14	NDRG2n; ARHGEF40ncd; ZNF219d	A	0.416	0.045	-0.003	0.012	0.795	-0.010	0.013	0.444	-0.043	0.032	0.182	-0.029	0.026	0.258	-0.021	0.029	0.482
Resting Heart Rate	rs12941356	17	SREBF1n; RAInd	G	0.181	0.033	0.021	0.009	0.014	0.019	0.009	0.038	0.042	0.022	0.056	0.036	0.018	0.048	0.021	0.020	0.290
Resting Heart Rate	rs13002735	2	B3GNT7nc	C	0.331	0.039	0.003	0.009	0.730	0.003	0.010	0.801	0.011	0.023	0.653	-0.019	0.020	0.329	0.026	0.020	0.185
Resting Heart Rate	rs13165531	5	CDH6n	T	0.221	0.034	0.003	0.008	0.717	0.001	0.008	0.906	-0.013	0.020	0.527	0.010	0.017	0.578	-0.012	0.017	0.481
Resting Heart Rate	rs1320761	6	GJA1n	C	0.902	0.053	-0.009	0.011	0.405	-0.009	0.012	0.412	0.010	0.027	0.702	-0.013	0.026	0.628	0.023	0.021	0.268

Resting Heart Rate	rs1468333	5	CDC23n	C	0.255	0.034	-0.011	0.008	0.177	-0.009	0.009	0.275	0.002	0.021	0.906	-0.027	0.018	0.125	0.001	0.017	0.946
Resting Heart Rate	rs1483890	3	FRMD4Bn	A	0.284	0.036	-0.009	0.009	0.344	-0.007	0.009	0.448	0.003	0.023	0.887	-0.001	0.019	0.949	-0.008	0.019	0.652
Resting Heart Rate	rs151041685	2	CCDC141ncd; TTNd	G	1.061	0.058	0.017	0.015	0.256	0.016	0.017	0.335	-0.035	0.042	0.405	-0.005	0.032	0.868			
Resting Heart Rate	rs1549118	14	ADCK1n	C	0.200	0.037	-0.009	0.009	0.292	-0.007	0.010	0.490	0.000	0.024	0.999	-0.006	0.019	0.774	0.015	0.020	0.469
Resting Heart Rate	rs16974196	19	C19orf47nd; MAP3K10e	G	0.244	0.036	0.010	0.009	0.263	0.013	0.009	0.165	0.026	0.022	0.245	0.018	0.020	0.355	-0.007	0.018	0.690
Resting Heart Rate	rs17180489	14	RGS6n	C	0.490	0.055	0.056	0.018	0.001	0.059	0.019	0.002	0.031	0.049	0.531	0.053	0.037	0.151			
Resting Heart Rate	rs17201923	14	FLRT2n	G	0.410	0.037	-0.015	0.010	0.114	-0.017	0.010	0.101	-0.020	0.027	0.452	-0.016	0.021	0.430	-0.004	0.023	0.859
Resting Heart Rate	rs17265513	20	ZHX3nc; EMILIN3d	T	0.240	0.042	0.007	0.012	0.525	-0.001	0.013	0.958	-0.073	0.032	0.021	0.004	0.025	0.861	-0.018	0.028	0.512
Resting Heart Rate	rs174536	11	MYRFn; FEN1e; FADS2e; TMEM258e	A	0.399	0.035	0.023	0.009	0.008	0.028	0.009	0.002	0.072	0.022	0.001	0.019	0.019	0.313	0.032	0.018	0.074
Resting Heart Rate	rs17881696	7	UFSP1nc; SRRTn; ACHEne; EPHB4d; GIGYF1d; PCOLCEd	G	0.578	0.043	0.000	0.011	0.993	0.004	0.012	0.739	-0.043	0.031	0.171	0.024	0.024	0.317	-0.010	0.028	0.716
Resting Heart Rate	rs180239	7	GNGT1n; GNG11n	C	0.326	0.035	-0.007	0.008	0.426	-0.009	0.009	0.296	0.001	0.022	0.959	-0.024	0.018	0.197	-0.018	0.018	0.338
Resting Heart Rate	rs1994135	12	SYT10n	T	0.400	0.033	0.006	0.008	0.495	0.013	0.009	0.164	0.016	0.022	0.454	0.048	0.018	0.006	-0.045	0.019	0.019
Resting Heart Rate	rs2076028	22	SUN2n; CBY1e; FAM227Ae; JOSD1e; TOMM22e; DDX17d; GTPBP1d	A	0.295	0.036	0.012	0.009	0.165	0.014	0.009	0.109	-0.005	0.021	0.832	0.033	0.019	0.078	-0.003	0.017	0.858
Resting Heart Rate	rs2152735	1	LMO4n	A	0.306	0.036	0.008	0.009	0.368	0.009	0.009	0.324	0.033	0.022	0.132	0.012	0.019	0.513	-0.020	0.018	0.276
Resting Heart Rate	rs2283274	12	CACNA1Cn	C	0.405	0.044	0.007	0.010	0.474	0.011	0.011	0.281	0.011	0.026	0.660	0.025	0.023	0.277	0.009	0.021	0.678
Resting Heart Rate	rs2358740	3	CACNA1Dn	T	0.208	0.035	0.001	0.008	0.921	0.001	0.009	0.947	-0.008	0.022	0.719	-0.017	0.019	0.354	-0.008	0.018	0.644
Resting Heart Rate	rs236349	6	PPIL1ne	A	0.281	0.035	-0.006	0.008	0.465	0.001	0.009	0.959	-0.009	0.021	0.672	0.013	0.019	0.512	0.001	0.017	0.973
Resting Heart Rate	rs272564	1	RNF220n	A	0.351	0.037	-0.014	0.009	0.107	-0.014	0.009	0.124	0.027	0.022	0.227	-0.019	0.019	0.336	-0.004	0.018	0.817
Resting Heart Rate	rs3749237	3	IP6K1n; GMPPBn; FAM212Ad; DAG1d;	G	0.258	0.035	0.021	0.009	0.021	0.018	0.010	0.061	0.044	0.023	0.058	0.008	0.019	0.663	0.032	0.020	0.107

			KLHDC8Bed; LAMB2d;																		
			PRKAR2Ad; QRICH1ed																		
Resting Heart Rate	rs3915499	16	MYH11nd	G	0.303	0.035	0.008	0.009	0.367	0.003	0.009	0.758	-0.023	0.022	0.292	-0.019	0.019	0.315	0.052	0.019	0.005
Resting Heart Rate	rs3951016	6	SCLC35F1n; PLNd	T	0.520	0.033	-0.004	0.008	0.647	-0.001	0.008	0.952	0.034	0.021	0.102	0.030	0.017	0.077	-0.005	0.018	0.798
Resting Heart Rate	rs41312411	3	SCN5And	G	0.320	0.047	-0.010	0.014	0.491	0.003	0.015	0.829	-0.028	0.034	0.419	0.004	0.029	0.882	0.009	0.028	0.759
Resting Heart Rate	rs41317993	1	CD46n; CD34d	G	0.630	0.055	-0.008	0.008	0.334	-0.004	0.009	0.629	0.004	0.021	0.861	-0.031	0.018	0.075	0.005	0.017	0.783
Resting Heart Rate	rs41748	7	METn	G	0.193	0.033	0.008	0.009	0.381	0.006	0.009	0.543	0.015	0.022	0.491	-0.017	0.019	0.364	0.035	0.019	0.072
Resting Heart Rate	rs422068	14	MYH6nd; MYH7d	T	0.731	0.034	0.004	0.008	0.612	0.001	0.009	0.953	-0.012	0.020	0.567	-0.009	0.017	0.614	-0.027	0.017	0.106
Resting Heart Rate	rs4608502	2	COL4A3n	T	0.249	0.035	0.002	0.015	0.887	0.022	0.017	0.176	0.014	0.041	0.742	-0.011	0.032	0.738			
Resting Heart Rate	rs4868243	5	NKX2-5n	A	0.361	0.044	0.011	0.009	0.184	0.011	0.009	0.221	0.031	0.022	0.163	-0.001	0.018	0.972	-0.031	0.019	0.107
Resting Heart Rate	rs4900069	14	C14orf159n	A	0.200	0.034	0.016	0.011	0.155	0.019	0.012	0.112	-0.012	0.030	0.704	0.029	0.024	0.216	0.001	0.027	0.976
Resting Heart Rate	rs4963772	12	SOX5n	A	0.714	0.047	-0.005	0.009	0.594	-0.008	0.009	0.377	0.011	0.023	0.617	0.003	0.018	0.894	-0.045	0.020	0.023
Resting Heart Rate	rs56233017	8	PLECn	A	0.666	0.083	-0.001	0.008	0.899	-0.002	0.009	0.861	0.010	0.021	0.643	0.009	0.018	0.613	-0.008	0.017	0.637
Resting Heart Rate	rs58437978	7	TBX20n	C	0.240	0.034	0.000	0.011	0.971	-0.006	0.012	0.587	-0.009	0.028	0.754	-0.090	0.025	0.000	0.039	0.022	0.081
Resting Heart Rate	rs6123471	20	KIAA1755nc	C	0.595	0.033	0.031	0.032	0.337	0.027	0.035	0.443	0.044	0.093	0.638	0.121	0.072	0.095			
Resting Heart Rate	rs61735998	18	FHOD3ncd	T	0.834	0.109	-0.009	0.011	0.420	-0.007	0.011	0.563	-0.004	0.028	0.875	-0.029	0.022	0.195	0.002	0.026	0.936
Resting Heart Rate	rs62172372	2	CALCRLne	A	0.337	0.042	0.011	0.009	0.244	0.016	0.010	0.104	0.017	0.024	0.474	0.071	0.020	0.000	0.018	0.020	0.345
Resting Heart Rate	rs6845865	4	ARHGAP10nd; EDNRAd	C	0.342	0.045	-0.028	0.009	0.001	-0.030	0.009	0.001	0.056	0.022	0.010	-0.102	0.019	0.000	-0.005	0.018	0.792
Resting Heart Rate	rs7194801	16	CDH11n	C	0.291	0.033	-0.009	0.008	0.263	-0.011	0.009	0.220	-0.028	0.021	0.183	-0.016	0.018	0.350	-0.021	0.018	0.256
Resting Heart Rate	rs73158705	7	CHRM2n	A	0.393	0.045	-0.007	0.010	0.522	-0.004	0.011	0.697	-0.016	0.025	0.532	0.000	0.023	0.987	-0.030	0.020	0.136
Resting Heart Rate	rs748802	9	HRCT1n	A	0.202	0.035	0.010	0.009	0.228	0.013	0.009	0.147	0.012	0.022	0.598	0.027	0.019	0.161	0.037	0.019	0.045
Resting Heart Rate	rs75190942	11	KCNJ5nd; C11orf45n	A	0.496	0.060	0.015	0.017	0.375	0.029	0.017	0.092	0.030	0.045	0.507	0.097	0.036	0.008	-0.015	0.038	0.699
Resting Heart Rate	rs7612445	3	GNB4n	T	0.428	0.042	0.005	0.010	0.641	0.013	0.011	0.225	-0.043	0.027	0.111	0.028	0.022	0.209	0.005	0.023	0.832
Resting Heart Rate	rs79121763	17	TEKT3n; PMP22d	T	0.471	0.063	0.006	0.019	0.763	-0.005	0.020	0.813	-0.013	0.051	0.795	-0.005	0.041	0.907			
Resting Heart Rate	rs867400	12	RASSF3nd	T	0.298	0.033	-0.003	0.008	0.690	0.000	0.009	0.962	-0.010	0.021	0.644	0.002	0.018	0.915	0.014	0.018	0.437
Resting Heart Rate	rs907683	2	SPEGnd; DESn	T	0.334	0.036	0.013	0.010	0.170	0.008	0.010	0.448	-0.009	0.025	0.709	0.061	0.021	0.004	-0.006	0.021	0.763

NT Pro-BNP	rs11105306	12	POC1B/GALNT4	C	0.200	0.183	0.001	0.010	0.895	0.003	0.011	0.798	-0.032	0.025	0.212	0.010	0.021	0.651	0.034	0.022	0.115
NT Pro-BNP	rs13107325	4	SLC39A8	T	0.240	0.208	-0.001	0.019	0.980	-0.006	0.021	0.787	0.021	0.053	0.697	-0.030	0.044	0.492			
NT Pro-BNP	rs198389	1	NPPB	G	0.425	0.152	0.019	0.008	0.026	0.019	0.009	0.039	0.064	0.022	0.004	-0.011	0.018	0.545	0.041	0.019	0.027

Supplement table 2: Causal effects of stroke and its subtypes on cardioembolic risk factors.

	AS		AIS		LAS		CES		SVS	
	BETS±SE	P	BETS±SE	P	BETS±SE	P	BETS±SE	P	BETS±SE	P
**Atrial Fibrillation**										
Simple median	0.148±0.020	$4.438*10^{-13}$	0.154±0.021	$3.601*10^{-13}$	0.103±0.052	0.048	0.664±0.045	$7.753*10^{-49}$	0.060±0.044	0.170
Weighted median	0.180±0.020	$4.328*10^{-19}$	0.192±0.022	$5.265*10^{-19}$	0.008±0.053	0.884	0.695±0.047	$6.598*10^{-49}$	0.032±0.045	0.470
MR Egger	0.155±0.029	$6.175*10^{-7}$	0.168±0.030	$2.562*10^{-7}$	-0.016±0.060	0.790	0.706±0.053	$3.454*10^{-24}$	-0.005±0.059	0.936
Inverse variance weighted	0.175±0.015	$7.745*10^{-30}$	0.183±0.016	$6.533*10^{-30}$	0.060±0.032	0.065	0.667±0.028	$8.810*10^{-125}$	0.010±0.032	0.756
(Intercept)	0.002±0.002	0.428	0.002±0.003	0.547	0.007±0.005	0.140	-0.004±0.004	0.386	0.001±0.005	0.769
**Myocardial Infarction**										
Simple median	0.169±0.036	$3.343*10^{-6}$	0.184±0.038	$1.446*10^{-6}$	0.382±0.090	$2.178*10^{-5}$	0.120±0.066	0.071	-0.019±0.082	0.822
Weighted median	0.180±0.035	$2.958*10^{-7}$	0.204±0.036	$1.283*10^{-8}$	0.382±0.093	$3.760*10^{-5}$	0.137±0.068	0.044	0.086±0.074	0.242
MR Egger	0.173±0.112	0.134	0.208±0.128	0.120	0.566±0.216	0.016	0.262±0.170	0.137	0.119±0.250	0.639
Inverse variance weighted	0.165±0.046	$3.169*10^{-4}$	0.166±0.053	0.002	0.396±0.090	$9.529*10^{-6}$	0.129±0.070	0.064	0.132±0.089	0.139
(Intercept)	-0.001±0.012	0.932	-0.005±0.014	0.724	-0.020±0.023	0.397	-0.015±0.018	0.399	0.001±0.025	0.956
**NT Pro-BNP**										
Simple median	-0.008±0.055	0.889	-0.027±0.058	0.638	0.099±0.179	0.580	-0.052±0.106	0.624	NA	NA
Weighted median	-0.042±0.045	0.352	-0.027±0.047	0.572	0.108±0.138	0.435	-0.011±0.086	0.901	NA	NA
MR Egger	0.422±0.315	0.408	0.351±0.339	0.489	2.229±1.009	0.271	-0.694±0.683	0.495	NA	NA
Inverse variance weighted	-0.056±0.041	0.169	-0.062±0.038	0.109	-0.084±0.201	0.675	-0.012±0.077	0.874	-0.228±0.085	0.007
(Intercept)	-0.082±0.054	0.369	-0.071±0.058	0.436	-0.396±0.172	0.260	0.117±0.116	0.498	NA	NA
**P-wave duration**										
Simple median	-0.011±0.006	0.087	-0.005±0.007	0.488	-0.006±0.014	0.677	-0.001±0.017	0.945	-0.001±0.013	0.943
Weighted median	0.000±0.006	0.951	0.003±0.006	0.608	-0.002±0.013	0.908	0.018±0.012	0.139	0.002±0.012	0.894
MR Egger	0.004±0.010	0.728	0.005±0.014	0.719	0.005±0.024	0.854	0.024±0.051	0.661	-0.005±0.031	0.885
Inverse variance weighted	-0.008±0.005	0.070	-0.006±0.006	0.363	-0.005±0.011	0.606	-0.012±0.022	0.600	-0.002±0.012	0.900

(Intercept)	-0.010±0.007	0.226	-0.009±0.011	0.423	-0.008±0.018	0.659	-0.029±0.038	0.472	0.003±0.023	0.915
**Resting Heart Rate**										
Simple median	0.011±0.005	0.049	0.007±0.006	0.246	0.028±0.014	0.051	0.028±0.013	0.035	-0.014±0.012	0.246
Weighted median	0.010±0.005	0.050	0.008±0.006	0.161	0.019±0.014	0.161	0.010±0.013	0.426	-0.013±0.011	0.239
MR Egger	0.009±0.010	0.373	0.009±0.011	0.388	0.030±0.023	0.188	0.012±0.025	0.635	-0.002±0.019	0.896
Inverse variance weighted	0.010±0.004	0.009	0.013±0.004	0.005	0.021±0.009	0.026	0.023±0.010	0.028	-0.009±0.007	0.203
(Intercept)	0.001±0.004	0.847	0.001±0.004	0.754	-0.004±0.008	0.656	0.004±0.009	0.637	-0.003±0.007	0.681
**PR interval**										
Simple median	0.000±0.002	0.873	-0.001±0.002	0.789	0.007±0.005	0.156	-0.001±0.004	0.839	-0.002±0.005	0.641
Weighted median	0.000±0.002	0.802	0.001±0.002	0.520	0.002±0.005	0.611	-0.001±0.004	0.749	0.007±0.004	0.065
MR Egger	0.000±0.004	0.986	0.002±0.004	0.694	-0.004±0.007	0.583	-0.009±0.009	0.334	0.013±0.007	0.094
Inverse variance weighted	0.000±0.002	0.942	0.000±0.002	0.829	0.000±0.003	0.972	-0.003±0.004	0.502	0.002±0.003	0.505
(Intercept)	0.000±0.004	0.982	-0.003±0.006	0.582	0.006±0.010	0.522	0.009±0.012	0.456	-0.016±0.010	0.122
**P-wave terminal force in lead V1**										
Simple median	0.000±0.000	0.300	0.000±0.000	0.501	0.000±0.000	0.105	0.000±0.000	0.865	0.000±0.000	0.170
Weighted median	0.000±0.000	0.464	0.000±0.000	0.484	0.000±0.000	0.181	0.000±0.000	0.651	0.000±0.000	0.140
MR Egger	0.000±0.000	0.799	0.000±0.000	0.799	0.000±0.001	0.925	0.000±0.000	0.405	-0.001±0.000	0.324
Inverse variance weighted	0.000±0.000	0.236	0.000±0.000	0.200	0.000±0.000	0.060	0.000±0.000	0.757	0.000±0.000	0.311
(Intercept)	-0.010±0.018	0.612	-0.011±0.019	0.599	-0.015±0.053	0.789	-0.036±0.039	0.425	0.044±0.041	0.393

AS: any stroke; AIS: any ischemic stroke; LAS: large artery stroke; CES: cardioembolic stroke; SVS: small vessel stroke.

Supplement figure1: Flowchart

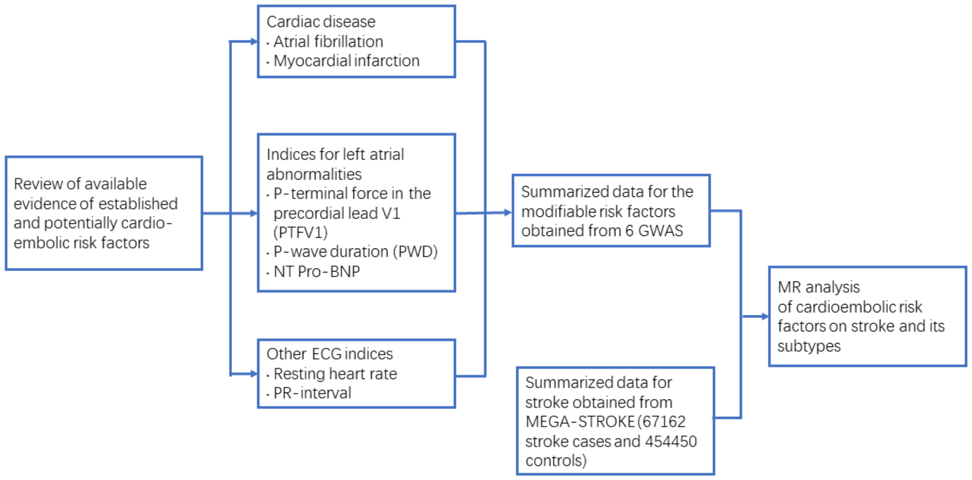

ECG: electrocardiograph, GWAS: genome wide association study, MR: Mendelian ransomization.

Supplement figure2: MR effect size for atrial fibrillation on any ischemic stroke (AIS).

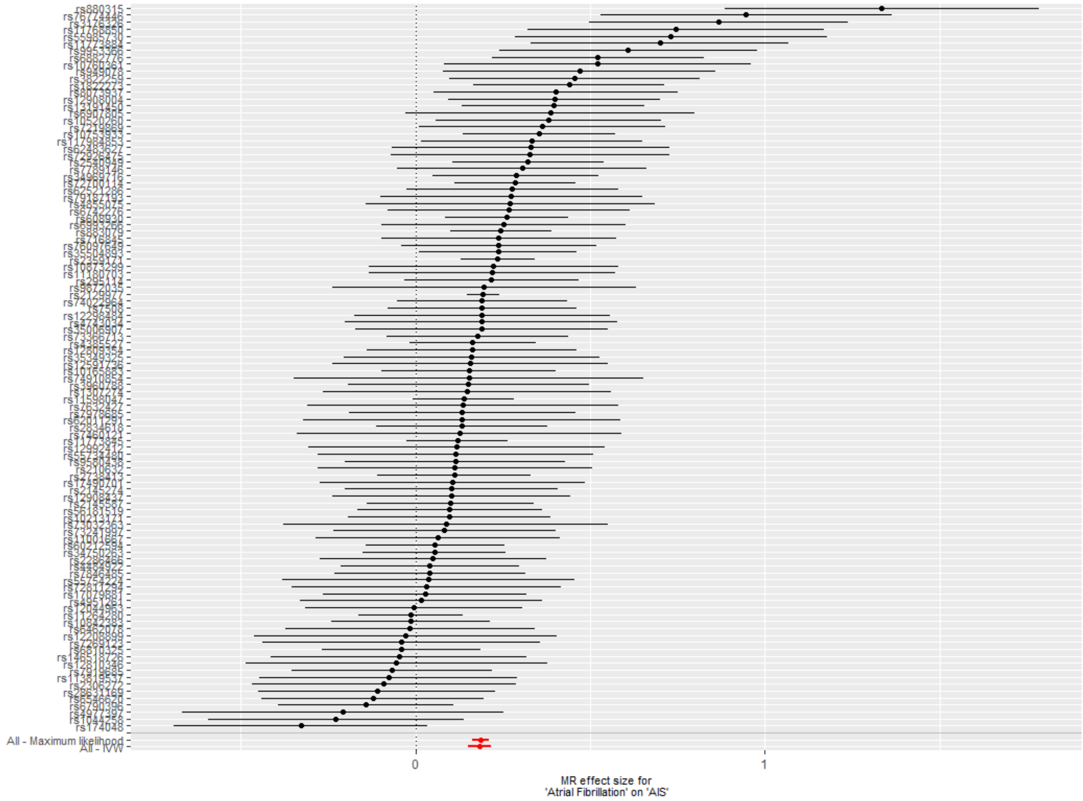

MR: Mendelian randomization.

Supplement figure3: MR effect size for atrial fibrillation on cardioembolic stroke (CES).

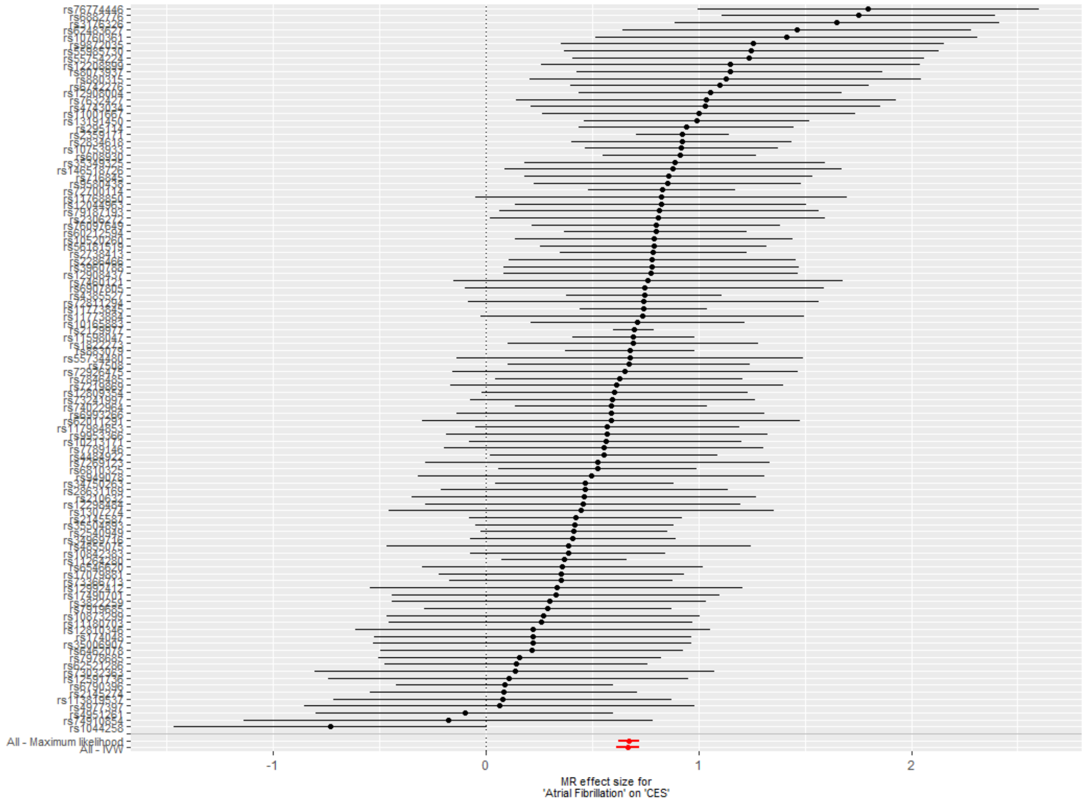

MR: Mendelian randomization.

Supplement figure4: Scatter plot for atrial fibrillation on any ischemic stroke (AIS).

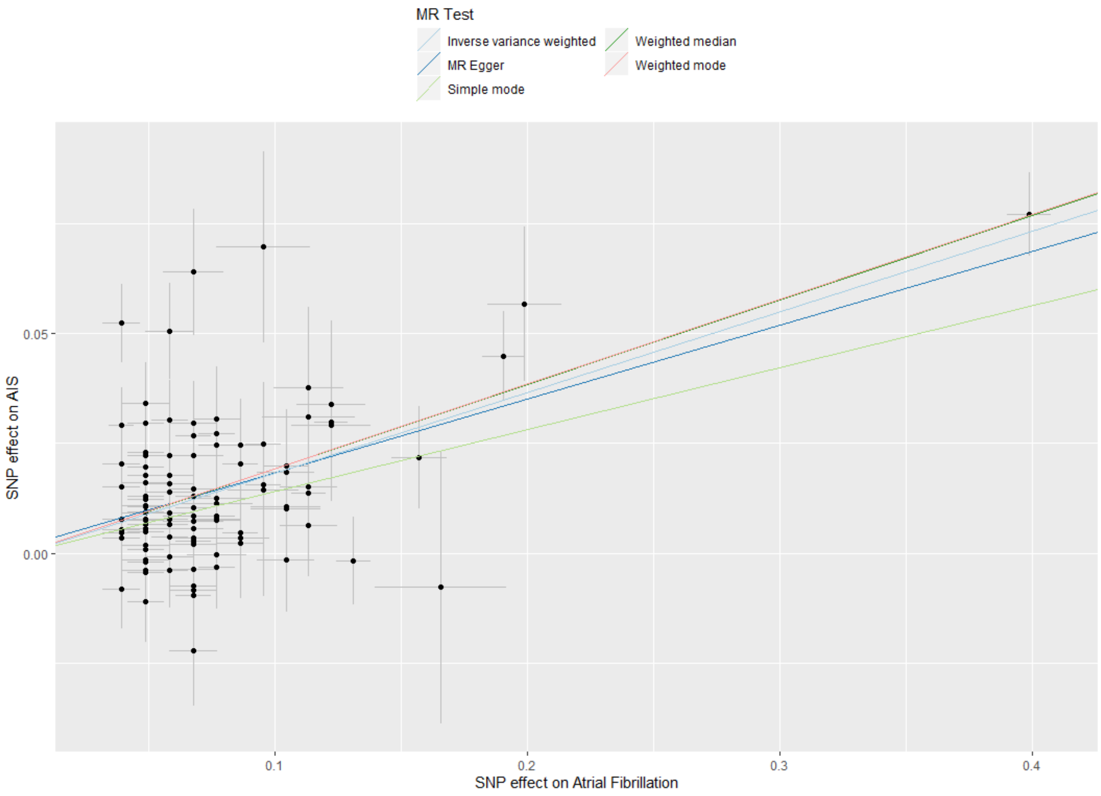

SNP: single nucleotide polymorphism

Supplement figure5: Scatter plot for atrial fibrillation on cardioembolic stroke (CES).

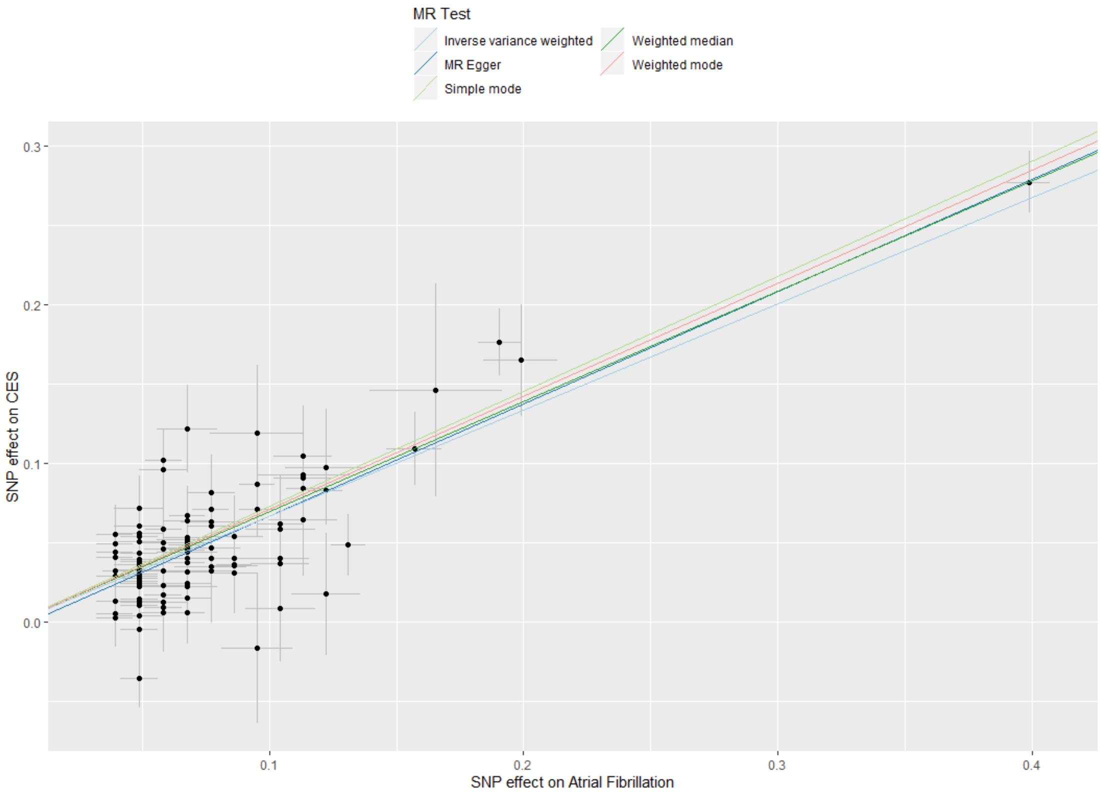

SNP: single nucleotide polymorphism,

Supplement figure6: MR effect size for myocardial infarction on any ischemic stroke (AIS).

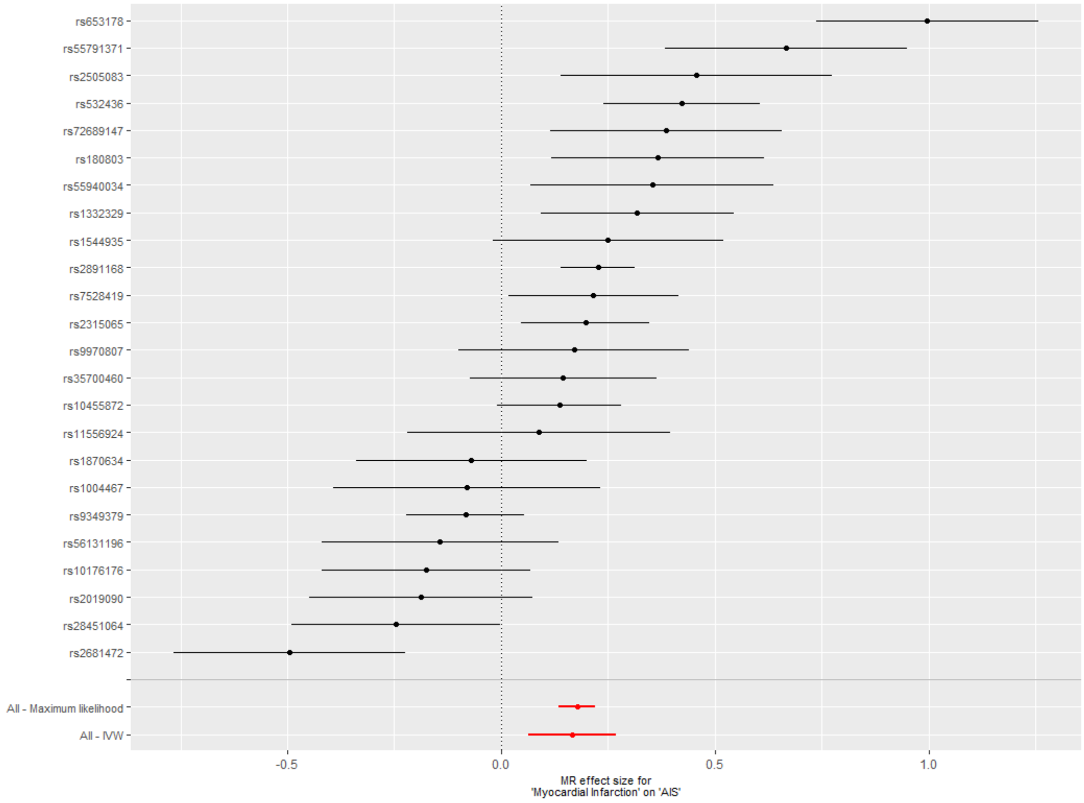

MR: Mendelian randomization.

Supplement figure7: MR effect size for myocardial infarction on large-artery atherosclerosis stroke (LAS).

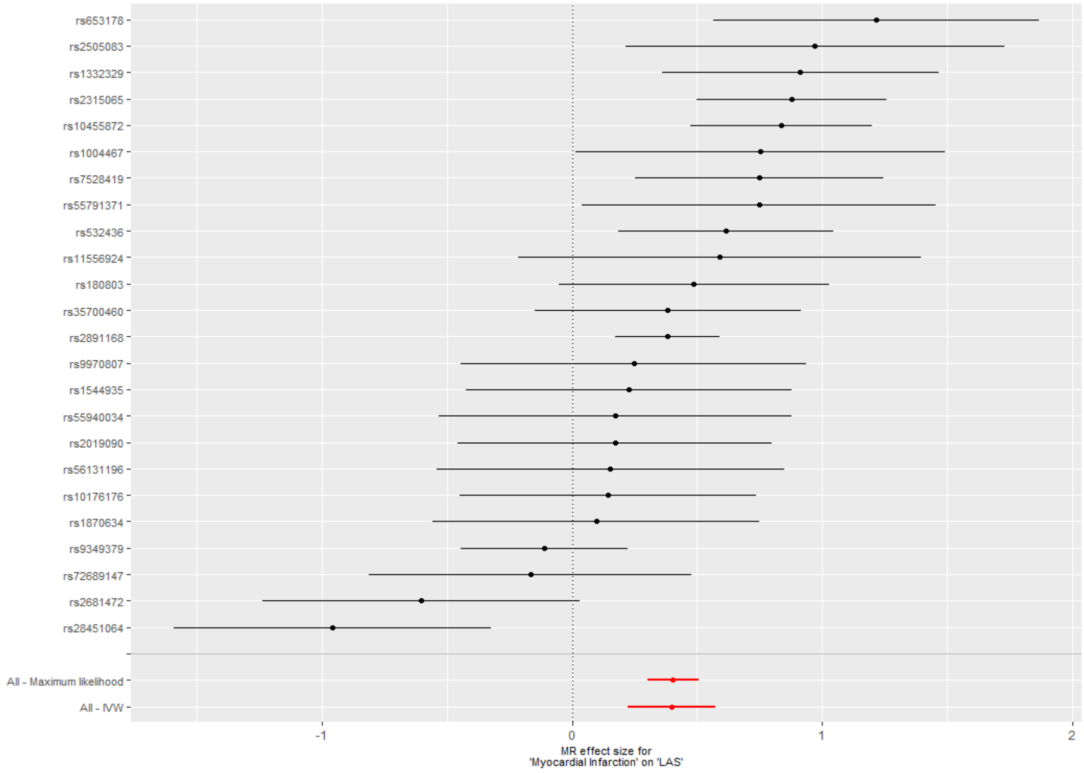

MR: Mendelian randomization.

Supplement figure 8: Scatter plot for myocardial infarction on any ischemic stroke (AIS).

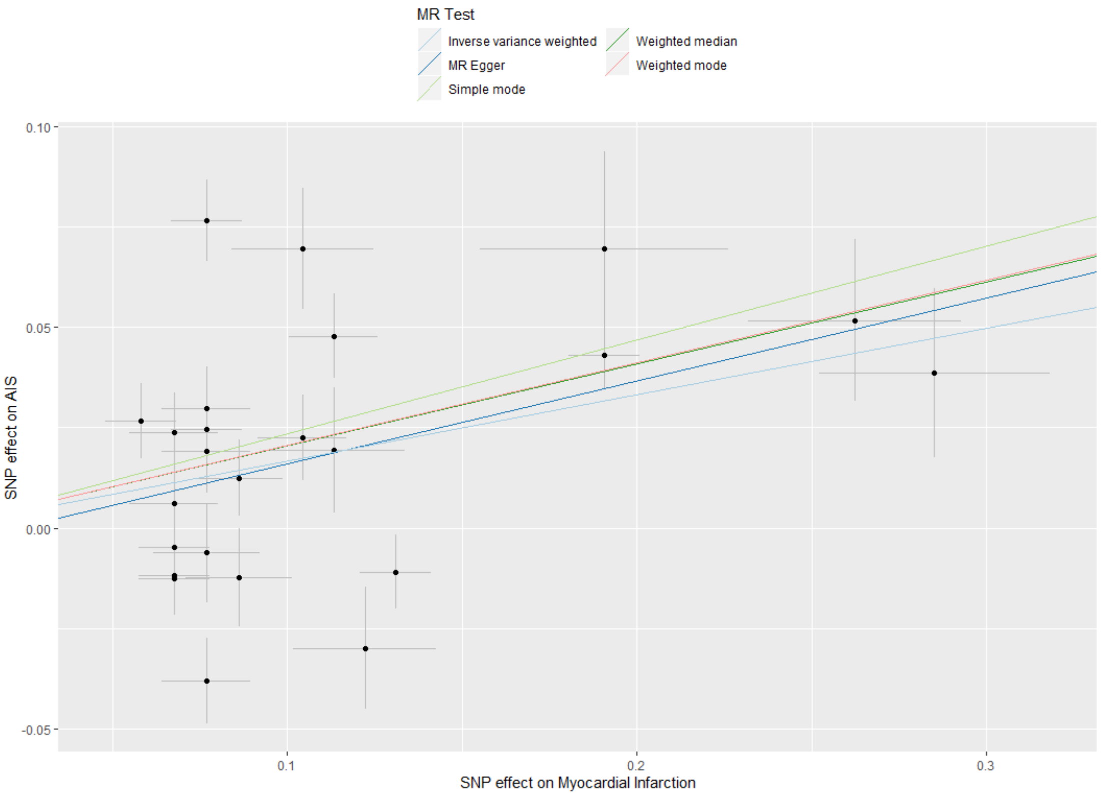

SNP: single nucleotide polymorphism,

Supplement figure 9: Scatter plot for myocardial infarction on large-artery atherosclerosis stroke (AIS).

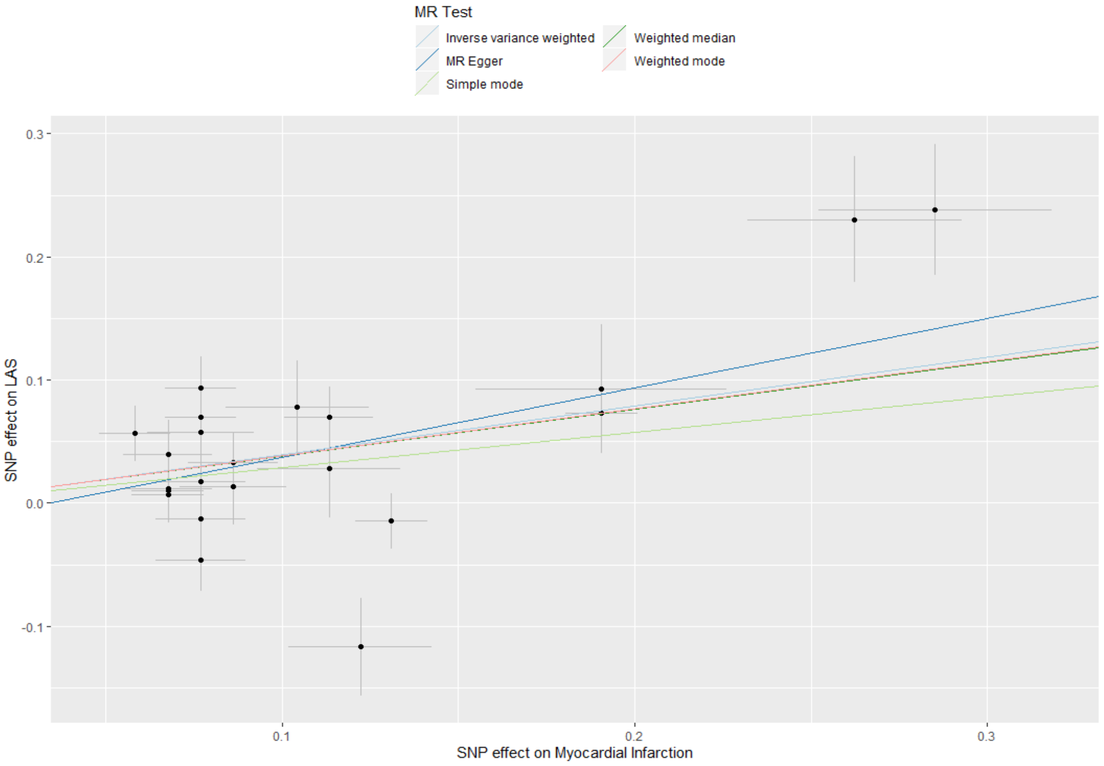

SNP: single nucleotide polymorphism,

Supplement figure10: leave-one-out analysis for myocardial infarction on cardioembolic stroke (CES).

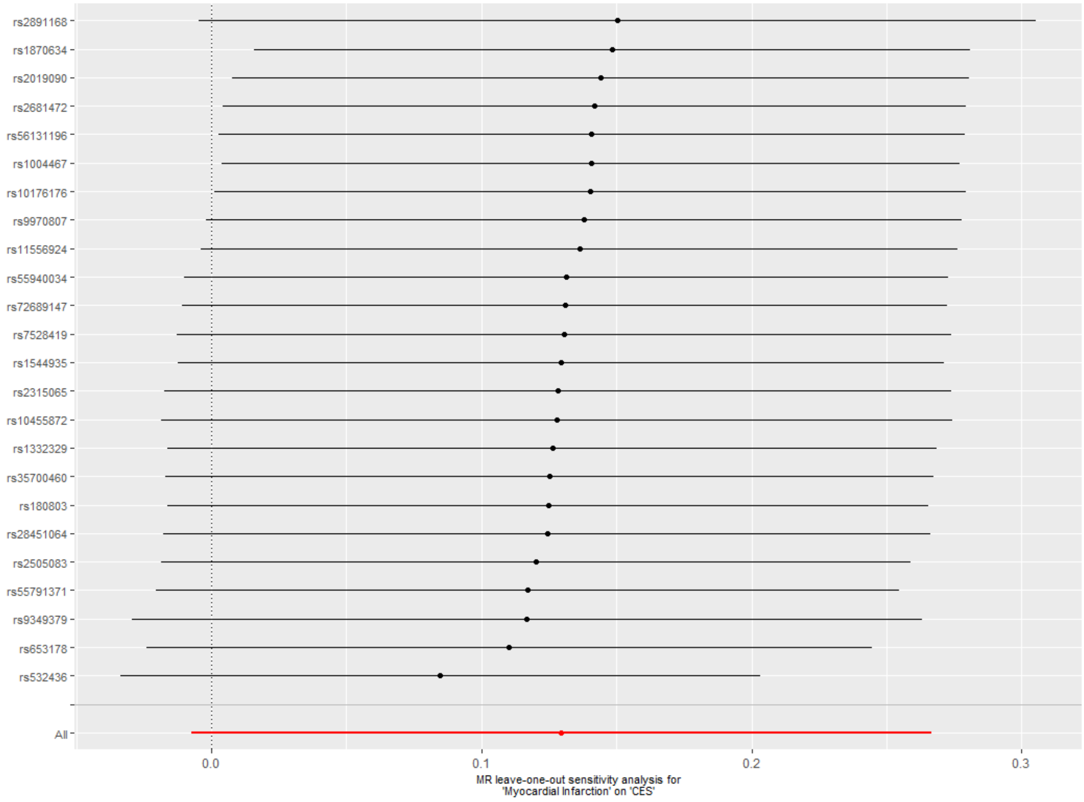

MR: Mendelian randomization.

Supplement figure 11: MR effect size for resting heart rate (RHR) on any ischemic stroke (AIS).

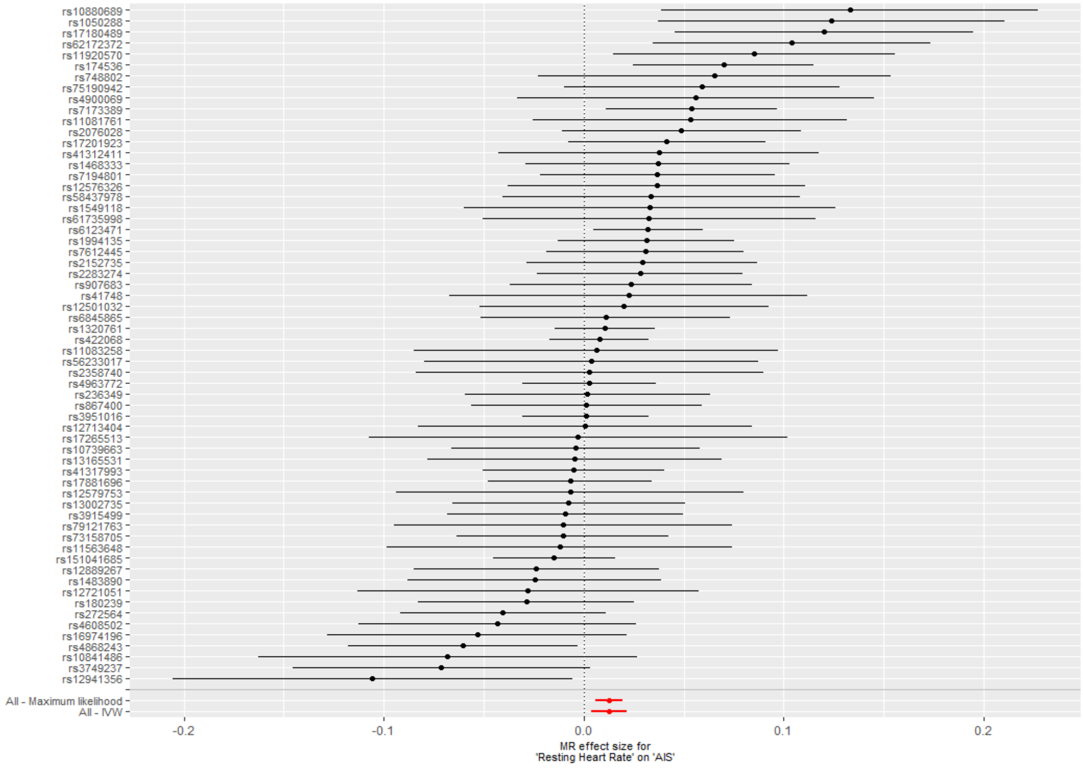

MR: Mendelian randomization.

Supplement figure 12: MR effect size for resting heart rate (RHR) on large-artery atherosclerosis stroke (LAS).

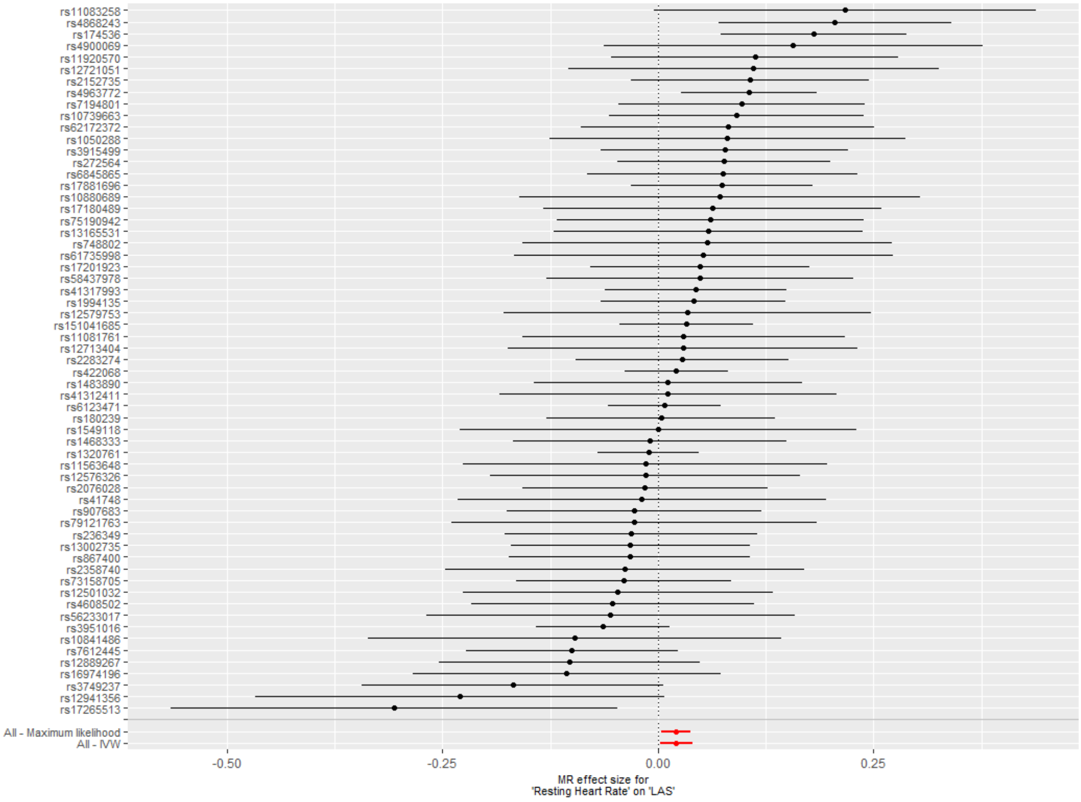

MR: Mendelian randomization.

Supplement figure 13: MR effect size for resting heart rate (RHR) on cardioembolic stroke (CES).

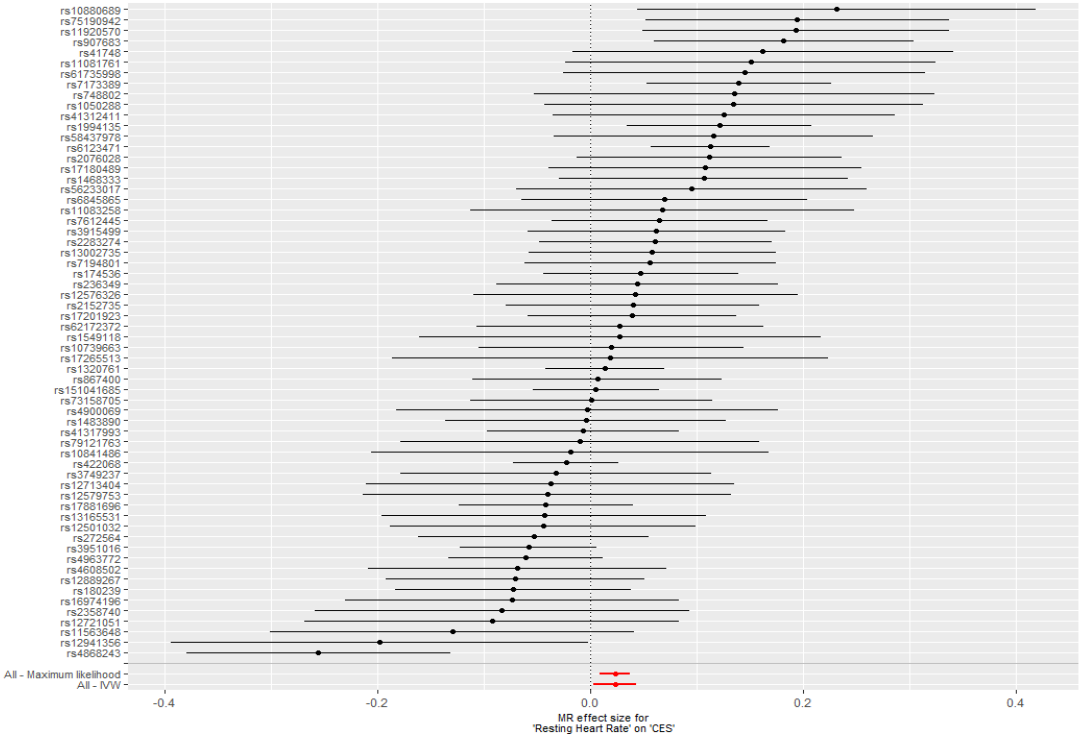

MR: Mendelian randomization.

Supplement figure14: Scatter plot for resting heart rate (RHR) on any ischemic stroke (AIS).

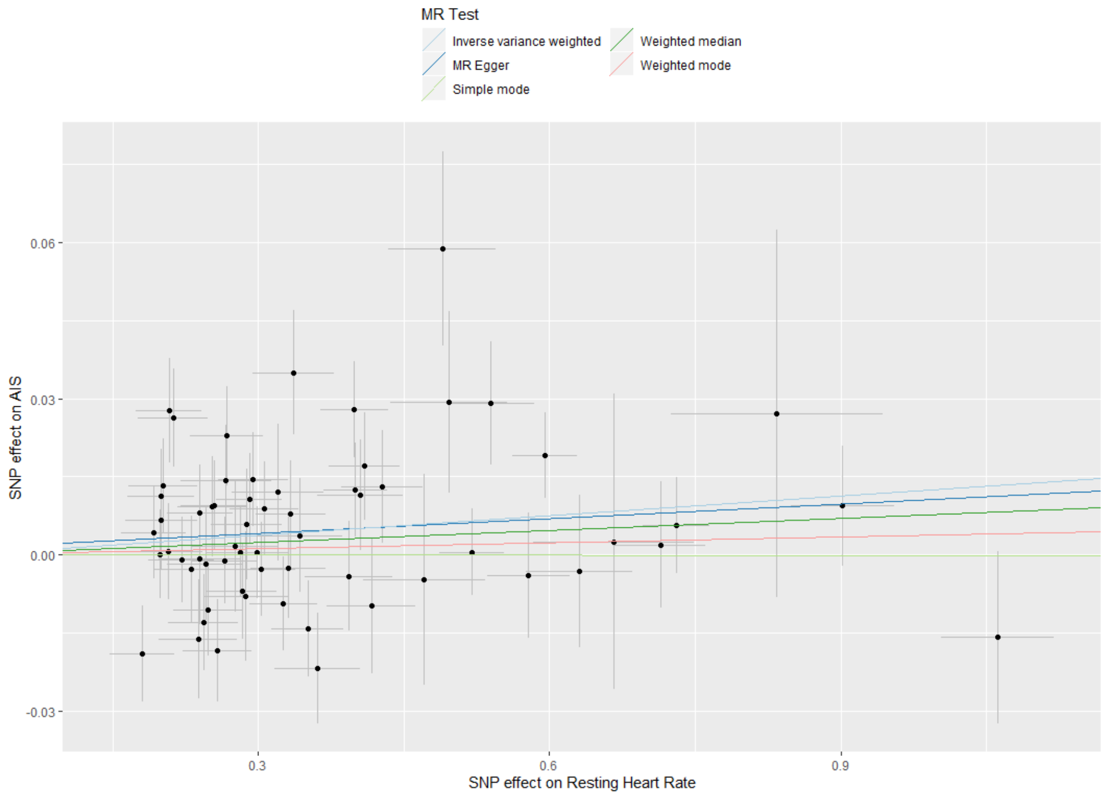

SNP: single nucleotide polymorphism,

Supplement figure 15: Scatter plot for resting heart rate (RHR) on large-artery atherosclerosis stroke (LAS).

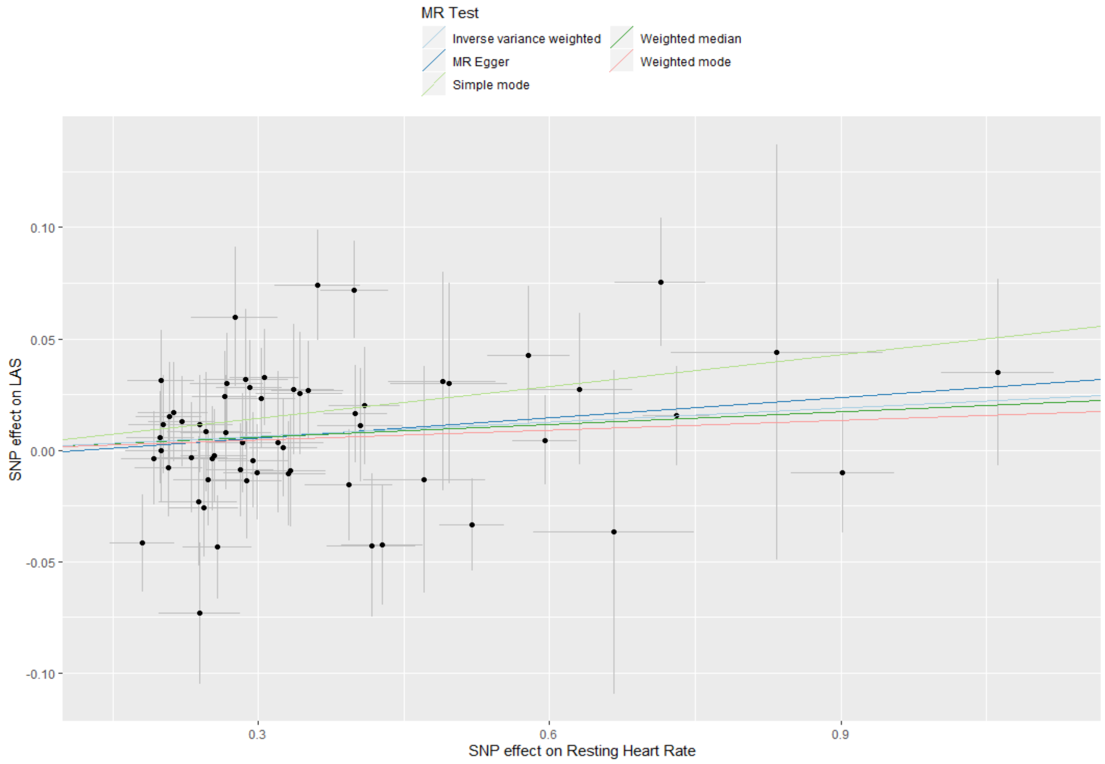

SNP: single nucleotide polymorphism,

Supplement figure16: Scatter plot for resting heart rate (RHR) on cardioembolic stroke (CES).

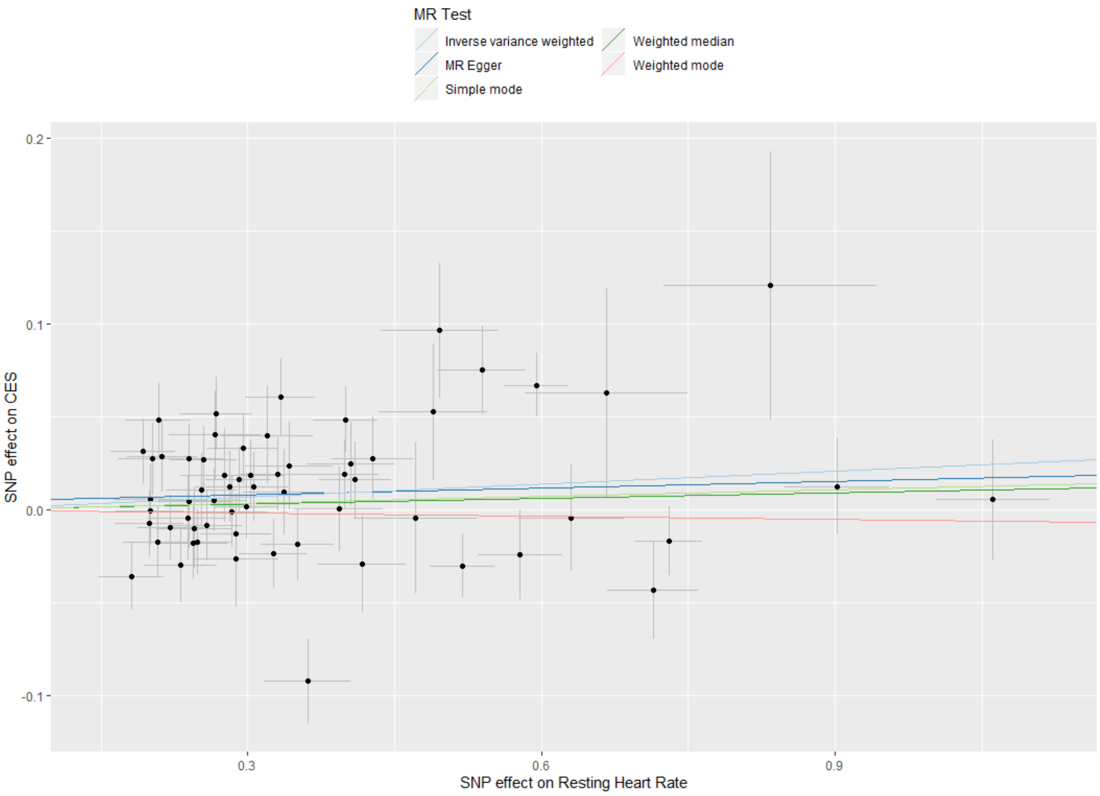

SNP: single nucleotide polymorphism,
